# Modeling sediment oxygen demand in a highly productive lake under various trophic scenarios

**DOI:** 10.1371/journal.pone.0222318

**Published:** 2019-10-09

**Authors:** Thomas Steinsberger, Beat Müller, Christoph Gerber, Babak Shafei, Martin Schmid

**Affiliations:** 1 Eawag, Swiss Federal Institute of Aquatic Science and Technology, Switzerland; 2 Institute of Biogeochemistry and Pollutant Dynamics, ETH Zürich, Switzerland; 3 Land and Water, Commonwealth Scientific and Industrial Research Organisation (CSIRO), Glen Osmond, SA, Australia; 4 AquaNRG Consulting Inc, Houston, Texas, United States of America; University of the Chinese Academy of Sciences, CHINA

## Abstract

Hypolimnetic oxygen depletion in lakes is a widespread problem and is mainly controlled by the sediment oxygen uptake (SOU) and flux of reduced substances out of the sediments (F_red_). Especially in eutrophic lakes, F_red_ may constitute a major fraction of the areal hypolimnetic mineralization rate, but its size and source is often poorly understood. Using a diagenetic reaction-transport model supported by a large data set of sediment porewater concentrations, bulk sediment core data and lake monitoring data, the behavior of F_red_ was simulated in eutrophic Lake Baldegg. Transient boundary conditions for the gross sedimentation of total organic carbon and for hypolimnetic O_2_ concentrations were applied to simulate the eutrophication and re-oligotrophication history of the lake. According to the model, F_red_ is dominated by methanogenesis, where up to70% to the total CH_4_ is produced from sediments older than 20 years deposited during the time of permanent anoxia between 1890 and 1982. An implementation of simplified seasonal variations of the upper boundary conditions showed that their consideration is not necessary for the assessment of annual average fluxes in long-term simulations. Four lake management scenarios were then implemented to investigate the future development of F_red_ and SOU until 2050 under different boundary conditions. A comparison of three trophic scenarios showed that further reduction of the lake productivity to at least a mesotrophic state is required to significantly decrease F_red_ and SOU from the present state. Conversely, a termination of artificial aeration at the present trophic state would result in high rates of organic matter deposition and a long-term increase of F_red_ from the sediments of Lake Baldegg.

## Introduction

O_2_ depletion in the hypolimnion of productive lakes and reservoirs still poses a challenge for their management. The sediment oxygen demand (SOD) combines the two main sinks, the sediment oxygen uptake (SOU, i.e. the diffusion-controlled flux of O_2_ to the sediment-water interface and its consumption within the top sediment layer) and the flux of reduced substances out of the sediment (F_red_) [1–3]. The SOU is dominated by the rapid aerobic mineralization of freshly deposited organic matter (OM) and contributions from the oxidation of reduced substances such as CH_4_ and NH_4_^+^. In lakes with a high nutrient load, O_2_ levels usually decrease to zero within the first millimeters of the sediment [4], while in oligotrophic lakes the O_2_ penetration depth can reach up to several centimeters [5]. The ensuing anaerobic degradation processes generate reduced substances such as CH_4_, NH_4_^+^, Mn(II), Fe(II) and S(-II), which once in contact with O_2_, will be oxidized. Hence, the combined fluxes of reduced substances (F_red_) from the sediment can be regarded as a negative O_2_ flux. Especially in the hypolimnion of shallow lakes, F_red_ can constitute the main sink for ambient O_2_ with up to 80% of the total hypolimnetic O_2_ consumption [2, 3].

Artificial aeration systems were installed in many anoxic lakes to increase hypolimnetic O_2_ levels. Surprisingly, SOD increased in the pursuit of artificial aeration, presumably because the additional O_2_ was consumed to mineralize large parts of recently deposited OM and to oxidize F_red_ generated by “legacy” OM [3, 6], which was largely buried during hypolimnetic anoxia. This phenomenon where sediments continue to be a major sink for DO, in spite of decades of artificial aeration, is called “sediment memory effect” [7]. However, it is still unclear under which circumstances hypolimnetic O_2_ depletion rates will start to decrease as the relative importance of SOU and F_red_ and their possible resilience to re-oligotrophication measures are poorly understood. To better understand these feedbacks and to navigate re-oligotrophication campaigns and aeration systems, a multi component early diagenesis model has been developed on the basis of the Van Cappellen and Wang [8], Couture, Shafei [9] and Dittrich, Wehrli [10] models. The model uses kinetic rate constants for biogeochemical reactions and processes coupled with a one-dimensional transport model using parameters mostly universal for the aquatic environment. This type of reaction network reflects the current state of the art of modeling [9–11] and enables the investigation of both O_2_ sinks, F_red_ and SOU. The model also allows simulations of the transient behavior of the O_2_ fluxes under varying boundary conditions due to seasonal variations or long-term trends such as eutrophication, re-oligotrophication or climate change.

This paper investigates the controls of long-term O_2_ depletion in the hypolimnion of the Swiss eutrophic Lake Baldegg. To this end, we model SOD of four scenarios for variations in total organic carbon (TOC) gross sedimentation rate and O_2_ concentrations, and depict the resulting fluxes of reduced substances out of the sediment and the flux of O_2_ into the sediments. The different modeling scenarios are based on sediment and sediment porewater data from Lake Baldegg. Lake monitoring data from Lake Baldegg was used to define the upper boundary conditions for the “Status Quo” (SQ) and “No artificial aeration” (NoAa) scenarios. Data from neighboring mesotrophic Lake Hallwil and from oligotrophic Lake Aegeri were utilized for the “Mesotrophic” and the “Oligotrophic” scenario, respectively.

In a first step, a model with constant boundary conditions was applied to reproduce average sediment porewater concentration. As SOD is governed by the quality and quantity of TOC deposited at the sediment surface and the overlying O_2_ concentrations [1, 2, 12], the different transient input parameters of the modeling scenarios show changes in SOD and help identifying lake restoration goals. Hence, in a second step, as lake primary production and other parameters such as hypolimnetic O_2_, NO_3_^-^ and SO_4_^2-^ concentrations and concentrations of reduced substances at the sediment-water interface (NH_4_^+^, Mn(II), Fe(II)) can show seasonal variations [13], their respective upper boundary concentrations were modeled with simplified cosine functions to assess the possible impact of seasonal variations on long term O_2_ depletion.

## Materials and methods

### Study site

The study was conducted with data from eutrophic Lake Baldegg located on the Swiss plateau. The lake with an area of 5.2 km^2^ has a maximum depth of 66 m, a mean hypolimnion depth of 27.6 m and water residence time of 4.3 years [14]. The catchment of 73 km^2^ is dominated by intensive agriculture and pig farms with a high surplus of manure. In 1982, the total phosphorus (TP) concentration reached 520 μgP L^-1^. The lake is still eutrophic today but has recovered to ∼25 μgP L^-1^. In spite of artificial aeration with O_2_ during the stratified season and forced circulation in winter, the bottom waters become sub- to anoxic towards the end of summer stagnation. Although P loads and TP concentrations have dramatically decreased over the last 35 years, primary production and TOC gross sedimentation rate did not show a downward trend [3]. At the deepest point, anoxic conditions as evidenced by the formation of varves occurred from 1885 until the start of the artificial aeration system in 1982 [15].

### Sediment data

We used a sediment porewater data set collected during two years of extensive field campaigns in 2014 and 2015. Sixteen sediment cores were retrieved for sediment porewater analysis (CH_4_, NH_4_^+^, Mn(II), Fe(II), SO_4_^2-^, NO_3_^-^, NO_2_^-^, dissolved inorganic phosphorus (DIP)) and several additional cores were taken for bulk sediment analysis such as dating (^210^Pb, ^137^Cs, varve counting), TOC content, total nitrogen (TN) total phosphorus (TP), and total sulfur and physical parameters such as water content and porosity [2]. The sediment cores were taken at the deepest part of the lake at 64 m water depth (47° 11´ 53´´ N, 8°15´36´´ E), four times during a course of a year (March, June, September, November). Details of the sediment core retrieval and analytical procedures are given in Steinsberger, Schmid [2].

Total Fe and Mn contents of the sediment were taken from Schaller, Moor [16]. Sediment trap material was collected bi-weekly and was analyzed for TN, TP and TOC gross sedimentation rates.

### Model formulation

The general model approach is outlined in Van Cappellen and Wang [8] and Couture, Shafei [9]. A system of partial differential equations corresponding to early diagenesis equations is automatically generated in MATLAB and solved by MATLAB’s built-in solver *pdepe*. The general, one-dimensional partial differential equation for the approximation of the temporal variations of the concentrations of soluble and solid substances in the sediments was used based on deposition, molecular diffusion, bioturbation and transformation processes [17]:

$$\frac{\partial (\varepsilon C_{i})}{\partial t}=\left[\frac{\partial }{\partial x}(D_{i}\varepsilon \frac{\partial (C_{i})}{\partial x})-\frac{\partial }{\partial x}(\vartheta \varepsilon C_{i})\right]+\sum \varepsilon r_{i}(x,t,C_{i},\ldots )$$ Eq 1

Here, *C*_*i*_ is the concentration of solid (μmol g^-1^) or solute (μmol cm^-3^) *i*, *x* is the positive downward position along the 1-D vertical domain (x = 0 corresponding to the sediment water interface (SWI)), and *t* is time. ∑εr_i_ (*z*,*t*,*C*_*i*_, *…*) the sum of the sources and sinks of all species *i*, including the rates of all biogeochemical reactions producing or consuming species, as well as the non-local transport processes that remove or add dissolved species, most notably bioturbation. ε = φ for solutes and ε = 1- φ for solids with φ defined as the sediment porosity. For solids, *D*_*i*_ is the bioturbation coefficient *D*_*b*_ (cm^2^ yr^-1^). For solutes, *D*_*i*_ is calculated as *D*_*b*_ + *D*_*sed*_ with *D*_*sed*_ = *D*_*mo*_ /(1-log(φ^2^) the tortuosity corrected molecular diffusion coefficient (*D*_*mol*_) at in situ temperature and salinity [18]. For solids and solutes, ϑequals to the burial velocity of the sediments (cm yr^-1^).

The upper boundary condition for solutes at the SWI (x = 0) is given by the flux through the diffusive boundary layer (DBL) above the SWI:

$$F_{i}^{upper}(t)=D_{mol}*\frac{B_{i}(t)-C_{i}(0,t)}{d_{DBL}}$$ Eq 2

Here, *B*_*i*_*(t)* is the concentration of the solute in the bottom boundary layer of the water column, and can be time-dependent in non-steady state simulations to reflect long-term or seasonal changes in bottom water concentrations with *d*_*DBL*_ as the thickness of the DBL. Potential geochemical reactions in the DBL are neglected.

For solid-bound species (e.g. total organic carbon or Fe/Mn solid species), the flux continuity condition at x = 0 is:

$$D_{b}\frac{\partial C_{i}}{\partial x}-\vartheta C_{i}=\frac{-J_{i}(t)}{\rho_{b}(1-\varphi )}$$ Eq 3

Where *J*_*i*_*(t)* is the (transient) depositional flux of any given solid-bound species. ρ_b_(1-φ) is the denominator ensuring consistency among the units of *J*_*i*_*(t)* (μmol cm^-2^ yr^-1^) and *C*_*i*_ (μmol g^-1^ for solid species) with ρ_b_ dry density (g cm^-3^).The depth dependence of the bioturbation coefficient *D*_*b*_ was approximated from Katsev and Dittrich [19]:

$$D_{b}=D_{b}^{0}\frac{1-\tanh\left(\frac{x-H}{\tau_{b}}\right)}{1-\tanh\left(-\frac{H}{\tau_{b}}\right)}+D_{bmin}$$ Eq 4

$D_{b}^{0}$ is the value at the SWI, τ_b_ shows the characteristic depth half interval in which most of the reduction of *D*_*b*_ occurs, while *H* is the depth of the steepest gradient of *D*_*b*_ in the sediment [20]. *D*_*bmin*_ is added to account for very small disturbances in the sediment and to increase numerical stability, however with a very low value (0.01 cm^-2^ yr^-1^).

At the lower boundary *x*_*L*_, zero gradients (equivalent to zero diffusive fluxes) are imposed for all solute and solid species:

$$\frac{\partial C_{i}(x_{L},t)}{\partial x}=0$$ Eq 5

The grid points between the sediment surface and the maximum simulated sediment depth (here 50 cm) were calculated using a curvature function. This function increases the number of grid points in the upper sediment where production/consumption rates are highest. In the top centimeter the resolution varied between 0.04 mm and 0.13 mm. In deeper less reactive sediments, fewer grid points were used to increase simulation speed with a resolution reaching 2.9 mm at 30 cm sediment depth and 4.8 mm at 50 cm sediment depth. Modeled time steps are automatically chosen by MATLABs PDE solver according to the chosen process rates, species concentrations and tolerance levels. The system of PDEs (partial differential equations) defined in section 2.3 is solved in Matlab using the function *pdepe* within the temporal *t*_*o*_ *< t < t*_*f*_ and spatial (*x*_*o*_ *< x < x*_*f*_) domains. The *pdepe* function is designed to solve initial-boundary value problems consisting of systems of parabolic and elliptic PDEs in space and time. The numerical method is based on a piecewise nonlinear Petrov-Galerkin method with second-order accuracy. This method solves the ordinary differential equations (ODE) from the spatial discretization of the PDEs, using the built-in Matlab ODE solver to obtain approximated solutions at specific times within a defined time interval. Furthermore, the Matlab code evaluates the boundary values at each time step separately and therefore enables transient boundary conditions. This feature becomes particularly useful when simulating the fate of compounds whose inputs are or can be changed by anthropogenic activity like artificial aeration or re-oligotrophication efforts e.g. a change in TOC gross sedimentation rate.

### Modeled species

Twelve solute and ten solid species are modeled as listed in Table 1. SOU is calculated as the flux of O_2_ into the sediment and F_red_ as the sum of the fluxes of reduced substances out of the sediment (NH_4_^+^, CH_4_, Mn(II), Fe(II), S(-II)). Based on the respective redox stoichiometry, the fluxes of reduced compounds (*J*_*x*_) were converted to fluxes of O_2_ equivalents and denoted in gO_2_ m^-2^ d^-1^ (Eq 6).

$$F_{red}=2*J_{CH4}+2*J_{NH4}+2*J_{S(-II)}+0.5*J_{Mn(II)}+0.25*J_{Fe(II)}$$ Eq 6

$$SOU=J_{O_{2}}$$ Eq 7

$$SOD=SOU+F_{red}$$ Eq 8

**Table 1:** Upper boundary conditions used for the simulated soluble and solid species in the various scenarios defined in section 2.8. For solutes, these are the concentrations in the bottom boundary layer above the DBL, for solids the depositional fluxes. Between the dates given here, values were linearly interpolated.

Time-dependent Input Parameters	1860	1890	1900	1982	1985	2018	SQ (2030)	M (2030)	O (2030)	NoAa (2030)
**O_2_ (μmol cm^-3^)**	0.180	10^−5^	10^−5^	10^−5^	0.1	0.1	0.1	0.15	0.2	10^−5^ (from 2020)
**NO_3_ (μmol cm^-3^)**	0.030	0.030	10^−5^	10^−5^	0.1	0.1	0.1	0.1	0.100	10^−5^
**SO_4_ (μmol cm^-3^)**	0.035	0.035	10^−5^	10^−5^	0.11	0.11	0.11	0.11	0.11	10^−5^
**NH_4_ (μmol cm^-3^)**	10^−4^	10^−4^	0.15	0.5	0.1	0.1	0.1	0.05	10^−4^	0.3
**Mn(II) (μmol cm^-3^)**	10^−10^	10^−10^	0.08	0.08	0.01	0.01	0.01	0.005	10^−10^	0.08
**Fe(II) (μmol cm^-3^)**	10^−10^	10^−10^	0.08	0.08	0.01	0.01	0.01	0.005	10^−10^	0.08
**S(-II) (μmol cm^-3^)**	10^−10^	10^−10^	0.03	0.034	10^−10^	10^−10^	10^−10^	10^−10^	10^−10^	0.03
**CH_4_ (μmol cm^-3^)**	10^−10^	10^−10^	0.1	0.8	0.05	0.05	0.05	0.05	10^−10^	100
**OM_1_ (μmol cm^-2^ yr^-1^)**	192.5	407.75	479.5	623 (from 1950)	623	623	623	350	210	623
**OM_2_ (μmol cm^-2^ yr^-1^)**	82.5	174.75	205.5	267 (from 1950)	267	267	267	150	90	267
**Mo**_**1**_ **(**μ**mol cm**^**-2**^ **yr**^**-1**^**)**	7	7	7	10	10	10	10	8	7	7
**Mo_2_(μmol cm^-2^ yr^-1^)**	1	1	1	1.25	1.25	1.25	1.25	1.25	1	1
**Foh_1_ (μmol cm^-2^ yr^-1^)**	18	18	18	23	23	23	23	20	18	18
**Foh_2_(μmol cm^-2^ yr^-1^)**	3.3	3.3	3.3	3.6	3.6	3.6	3.6	3.4	3.3	3.3
**D_bo_ (Bioturbation)**	1	1	0	0	0.2	0.7	0.7	1	1	0
**Sedimentation rate (cm yr^-1^)**	0.15	0.15	0.18	0.33 (from 1950)	0.33	0.33	0.33	0.22	0.15	0.33
**Constant Input Parameters**										
**CO_2_ (μmol cm^-3^)**	0.01									
**HCO_3_ (μmol cm^-3^)**	1									
**N_2_ (μmol cm^-3^)**	0.1									
**HPO_4_ (μmol cm^-3^)**	0.001									
**FeS (μmol cm^-2^ yr^-1^)**	10^−10^									
**Pyrite (μmol cm^-2^ yr^-1^)**	10^−10^									
**Vivianite (μmol cm^-2^ yr^-1^)**	10^−5^									

Both measured and modeled porewater fluxes were calculated from the vertical porewater concentration gradients by Fick’s first law. Measured porewater fluxes were further evaluated by a one-dimensional reaction-transport model [21] that was adapted from Epping and Helder [22].

Although many studies use three OM pools (reactive, slow reactive and non-reactive) [10, 19], this model uses only two OM pools (OM_1_, reactive; and OM_2_, non-reactive). While the total amount of settled organic matter or TOC can be accurately determined, the ratio of reactive to non-reactive TOC is not known. After setting the main reaction rates, the ratio of reactive to non-reactive TOC was determined by comparing simulated and measured CH_4_, NH_4_,TOC and TN concentrations. Best results were found for 2/3 reactive and 1/3 non-reactive TOC. Similarly, the boundary conditions of total Fe and Mn were estimated from sediment trap analyses assuming that the predominant fraction of these metals is reactive (F_oh1_, 82% of total Fe; and M_o1_ 63% of total Mn), while a smaller part is less reactive (F_oh2_, 13% of total Fe; and M_o2_ 8% of total Mn) with the remaining fraction being non-reactive and consequently not modeled. The relative fractions of reactive and non-reactive Fe and Mn were calibrated to match the measured Fe(II) and Mn(II) porewater profiles and total solid Fe and Mn sediment profiles measured by Schaller, Moor [23]

The input of both organic matter pools is characterized by the measured elemental C (cx1): N (cy1): P (cz1): S (cs1) ratio of 106: 8: 0.5: 0.5. Other elements contained in the OM are discarded. Hence, by the turnover of one OM_1_ 106 carbon (cx1), 8 nitrogen (cy1), 0.5 phosphorous (cz1) and 0.5 sulfur (cs1) are liberated (see S1 Table).

### Seasonal modeling

**Fig 1:**
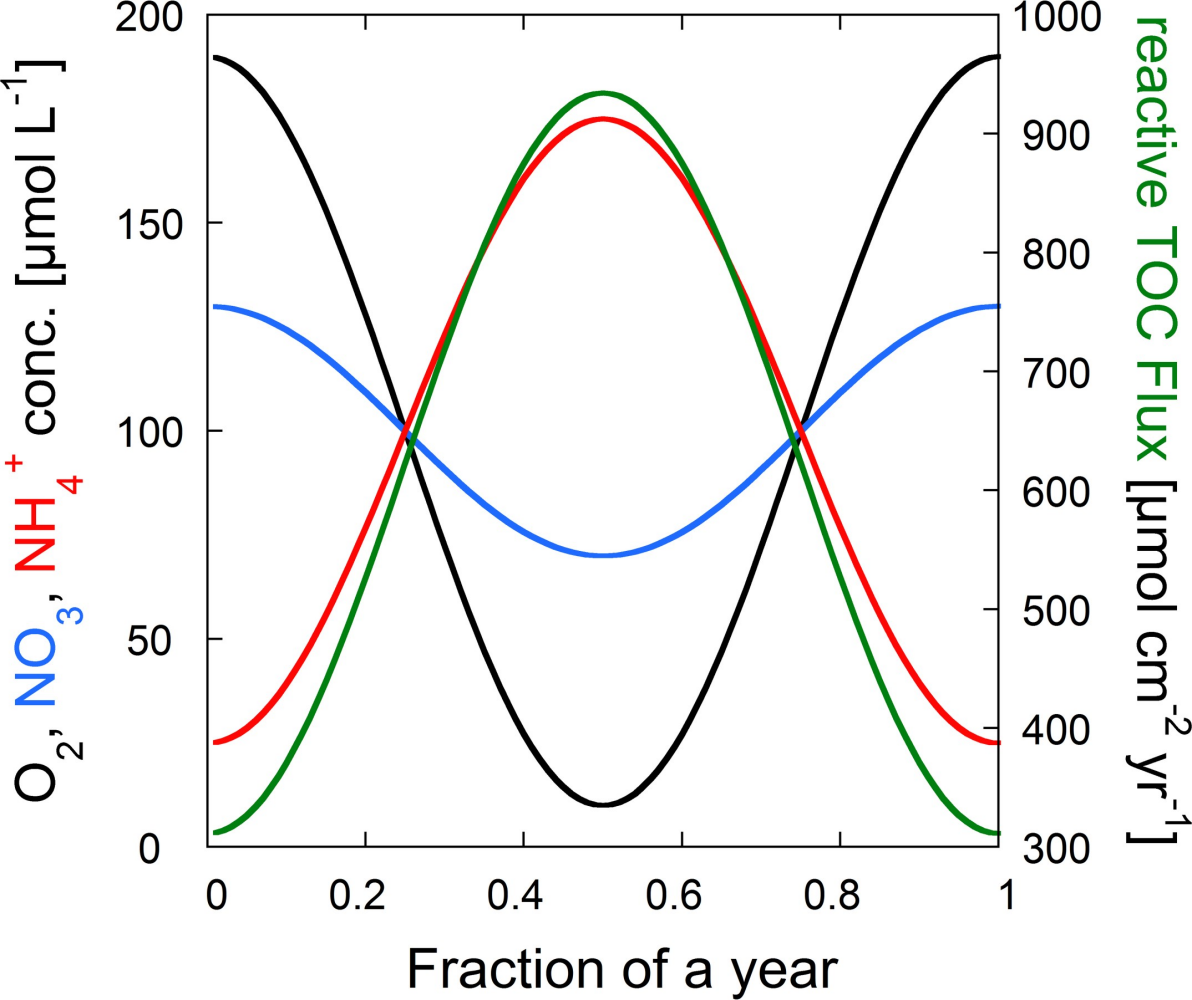
Simplified seasonal variations in the boundary concentrations of the “status quo” scenario shown for selected TEAs, the reactive TOC flux and NH_4_^+^.

Seasonal changes of TOC gross sedimentation rate and concentrations in O_2_, NO_3_^-^, SO_4_^2-^, NH_4_^+^, CH_4_, Fe(II), Mn(II) were observed during the two year-long field campaign [2]. These naturally occurring seasonal variations could influence the hypolimnetic O_2_ depletion rate. As the model allows for temporally varying upper boundary conditions, seasonal variations can be readily appended. However, direct data inputs of e.g. highly fluctuating TOC gross sedimentation rates captured in a daily and/or biweekly basis, result in numerical instability. Instead, the boundary conditions were multiplied with cosine functions with different amplitudes and phase shifts as simple approximations for the measured seasonal fluctuations (see Fig 1 and Table 2)[10]. The same cosines functions were applied to all scenarios leading to slight overestimations of hypolimnetic O_2_ levels in the “oligotrophic scenario”.

**Table 2:** Seasonal variations in boundary concentration for a list of modeled species calculated by various cosines functions. For the seasonal model, the boundary conditions from in Table 1 were multiplied with the seasonal functions given here. t is the time in years.

Changing Input Parameters	Seasonal variation function
O_2_	1+0.9xcos(2xπxt)
NO_3_	1+0.3xcos(2xπxt)
SO_4_	1+0.1xcos(2xπxt)
NH4, Mn(II), Fe(II), CH_4_	1–0.75xcos(2xπxt)
OM_1_, OM_2_, Mo_1_, Mo_2_, Foh_1_, Foh_2_	1–0.5xcos(2xπxt)

### Biogeochemical reactions

All biogeochemical reactions are listed in S2 Table. The mineralization processes are calculated with the stoichiometric coefficients of the OM cx_1_ (C), cy_1_ (N), cz_1_ (P) and cs_1_ (S). The biogeochemical degradation of OM is cascading according to the standard electron acceptor utilization (O_2_, NO_3_^-^, Mn-oxides, Fe-(hydr)oxides, SO_4_^2-^, and terminal methanogenesis) based on the available Gibbs Free energy [24]. Hence, the decomposition of the OM_1_ pool and availability of terminal electron acceptors (TEA) fundamentally drive all reactions considered in this model.

For this study, the concentrations and fluxes of O_2_ and NH_4_^+^, CH_4_, Mn(II), Fe(II) and S(-II) are of particular importance as they compose SOU and F_red_. Other secondary reactions and mineral precipitation and dissolution reactions are included and act as sources or sinks for reduced substances. NH_4_^+^ is released in each primary mineralization. Once O_2_ concentrations decline sufficiently, denitrification becomes the dominant mineralization process, where N_2_ is produced and lost to the overlying water and is not further modeled. Once NO_3_ is sufficiently depleted, Mn-oxides, Fe-(hydr)oxides and SO_4_^2-^are utilized as electron acceptors and reduced to Fe(II), Mn(II) and S(-II). During the final methanogenesis, NH_4_^+^, CH_4_ and CO_2_ are produced. All reduced substances are prone to aerobic oxidation by secondary reactions e.g. nitrification or anaerobic oxidations such as anaerobic methane oxidation (AMO) or oxidation by other oxides such as ferric iron. Further, various more stable forms of Fe- and Mn-containing mineral phases are formed such as iron sulfides, pyrite, vivianite, and manganese carbonates.

### Simulated scenarios

The model and the respective modeling scenarios are based on historic observations, lake monitoring data, bulk sediment parameters and sediment porewater data of Lake Baldegg [2, 23, 25, 26]. To calculate the different scenarios, two other lakes of comparable size and morphometry were chosen where complete limnological data sets were available. Similar to Lake Baldegg, neighboring Lake Hallwil was aerated since the 1980s but already shows decreased primary production and was therefore chosen as representative for the mesotrophic scenario. Lake Aegeri, where total phosphors concentrations are below 10 μg L^-1^ due to the low fraction of agriculture in its catchment, was chosen as representative for the oligotrophic scenario. The initial conditions were set to match the presumed natural oligotrophic state of the lake with average DO concentrations of 5.76 mg O_2_ L^-1^ available at the sediment water interface and low TOC gross sedimentation rate and were modeled for 350 years. All boundary conditions can be found in Table 1 and all reaction rates in Table 3. The transient boundary conditions of the model were then used to simulate the advancing eutrophication of Lake Baldegg starting in 1860 with decreasing O_2_ concentrations until permanent anoxic conditions were reached in 1890. These anoxic conditions are preserved in the sediments by the onset of sediment varves [15, 25]. The anoxic phase in the hypolimnion of Lake Baldegg lasted at least from 1890 until 1982 and was only reversed by the initiation of artificial aeration. During the anoxic phase, the boundary conditions of all reduced substances, especially for NH_4_^+^, rose as they started accumulating in the hypolimnetic waters and became an increasing environmental problem. Furthermore, this condition led to enhanced geochemical focusing as more reduced manganese and iron were exported to the sediments of the hypolimnion [27]. Based on the available lake O_2_ monitoring data, following the onset of aeration boundary O_2_ concentrations were set to 3.2 mg O_2_ L^-1^ between 1982 and 2018. The simulations were then continued from 2018 to 2050 with four scenarios with different upper boundary conditions to represent different trophic and oxic conditions in Lake Baldegg until the year 2050:

1. **Status Quo (SQ)** scenario, in which O_2_ concentrations (average 3.2 mg O_2_ L^-1^) and TOC gross sedimentation rates (107 g C m^-2^ yr^-1^) remain constant at current levels.
2. **Mesotrophic production (M)** scenario based on lake monitoring data from neighboring mesotrophic Lake Hallwil [2], which has been undergoing a re-oligotrophication phase since the early 1980s. Similar to Lake Baldegg, lake TP concentrations in Lake Hallwil decreased from 235 μg P L^-1^ in 1976 to 90 μg P L^-1^ in the 1990s and further to 12 μg P L^-1^ in 2015. However in Lake Hallwil, this change was accompanied by a drastic decrease of primary production and hence TOC gross sedimentation from over 100 g C m^-2^ yr^-1^ [28] to around 60 g C m^-2^ yr^-1^ [2]. Hence, for the M scenario the TOC gross sedimentation rate was reduced by 44% of the present day value and O_2_ concentrations were increased to an annual average of 4.8 mg O_2_ L^-1.^as observed in Lake Hallwil.
3. **Oligotrophic production (O)** scenario: Here we assume that TP concentrations rapidly decline over a 12-year period to a low productive state with low TOC gross sedimentations rates. Sediment trap data (TOC gross sedimentation) and O_2_ measurements from oligotrophic Lake Aegeri were used as proxy values for Lake Baldegg in a low productive state. In 2013 and 2014, in Lake Aegeri TOC gross sedimentation rates were 36 g C m^-2^ yr^-1^ [2] and hence the TOC flux in this scenario is reduced to 33% of the present day value. The O_2_ concentration value was set to an annual average of 6.4 mg O_2_ L^-1^.
4. **No artificial aeration scenario (NoAa):** As artificial aeration systems are expensive due to maintenance costs, the reduction of maintenance costs or termination of the aeration systems are regularly debated. However, it is highly likely that without artificial aeration Lake Baldegg would rapidly turn anoxic during summer stagnation, and depending on the intensity of winter mixing, most probably become permanently anoxic within a few years, similar to its status before the onset of artificial aeration in 1982. Therefore, the O_2_ concentration is set to 0 mg L^-1^. Similarly, NO_3_^-^ and SO_4_^2-^ concentrations are decreased. The TOC gross sedimentation is assumed to be not affected by this and is kept constant at the present-day level. Furthermore, the boundary conditions of the reduced substances NH_4_^+^, CH_4_, Fe(II), Mn(II) and S(-II) are increased to 300 μmol L^-1^, 100 μmol L^-1^, 80 μmol L^-1^, 80 μmol L^-1^and 30 μmol L^-1^_,_ respectively, to account for their likely accumulation in the hypolimnion (see Table 1).
  The underlying premise of the SQ, M and O scenarios is that artificial aeration remains active to supply sufficient hypolimnetic DO. The detailed boundary conditions for each scenario are given in Table 1. Finally, all scenarios were modeled with simplified seasonal variations of the TOC gross sedimentation rate, the boundary concentration of all TEAs (O_2_, NO_3_^-^, Mn(IV)-oxides, Fe(III)-hydroxides, SO_4_^2-^) and the boundary concentrations of all reduced substances.

**Table 3:** Model parameters used in the model. The reaction rates and corresponding reactions are defined in the S1 and S2 Tables. The asterisks are used to distinguish between calibrated (*), literature (**) or measured (+) parameters.

Parameter	Description	Value	Units	Literature values.	Refs.
Simulated sediment depth (+)		50	cm		
Sediment density (+)		2.48	g cm^-3^	2.5	[19]
Sedimentation rate (*)		0.15	cm yr^-1^		
Bioturbation coeff. ($D_{b}^{0}$) (**)		1	cm^2^ yr^-1^	0.1–5	[19]
*H*_*bio*_ (**)	Depth of max. bioturbation gradient	0.5	cm		[19]
*τ*_*bio*_ (**)	Bioturbation depth attenuation	1.43	cm	1–3	[19]
Dbl (**)	Diffusive boundary layer thickness	0.082	cm	0.082	[1, 3]
Porosity(*)		0.84			
$k_{deg,O_{2}}$ (**)	Oxic respiration	9.1	yr^-1^	9.13	[10]
$k_{deg,{NO}_{3}}$ (**)	Denitrification	7.3	yr^-1^	7.31	[10]
$k_{deg,{MnO}_{2}}$ (*)	MnO_2_ reduction	1.08 x 10^−2^	yr^-1^	3.65 x 10^−2^	[10]
*k*_*deg*,*FeOOH*_ (*)	FeOOH reduction	1.08 x 10^−2^	yr^-1^	1.83 x 10^−4^	[10]
$k_{deg,{SO}_{4}}$(*)	Sulfate reduction	1.44	yr^-1^	3.65 x 10^−2^	[10]
$k_{deg,{CH}_{4}}$(*)	Methanogenesis	5.28 x 10^−2^	yr^-1^	5.84 x 10^−3^	[10]
$k_{deg,O_{2},inh}$ (*)	Rate inhibition concentrations	1 x 10^−3^	μmol cm^-3^	10−4–10^−2^	[8, 19]
$k_{deg,{NO}_{3},inh}$ (*)		1 x 10^−3^	μmol cm^-3^	10−3–10^−1^	[19, 29]
$k_{deg,{MnO}_{2},inh}$ (*)		16	μmol g^-1^	16–200	[8, 19]
*k*_*deg*,FeOOH,*inh*_ (*)		100	μmol g^-1^	3–2000	[8, 19]
$k_{deg,{SO}_{4},inh}$ (*)		0.1	μ mol cm^-3^	0.06–1	[19]
*k*_*nhox*_ (*)	NH_4_ oxidation by O_2_	500	cm^3^ μmol^-1^ yr^-1^	5000	[19]
*k*_*mox*_ (*)	Mn(II) oxidation by O_2_	7500	cm^3^ μmol^-1^ yr^-1^	800–2000	[8]
*k*_*fox*_ (*)	Fe(II) oxidation by O_2_	1 x 10^4^	cm^3^ μmol^-1^ yr^-1^	3.5 x 10^4^	[19]
*k*_*sox*_ (*)	S(-II) oxidation by O_2_	160	cm^3^ μmol^-1^ yr^-1^	160–16000	[19]
*k*_*chox*_ (*)	CH_4_ oxidation by O_2_	3 x 10^5^	cm^3^ μmol^-1^ yr^-1^	1 x 10^7^	[8]
*k*_*nhmo*_ (*)	NH_4_ oxidation by reactive MnO_2_	7.5 x 10^−4^	cm^3^ μmol^-1^ yr^-1^	0	[19]
*k*_*nhmx*_ (*)	NH_4_ oxidation by less-reactive MnO_2_	7.5 x 10^−5^	cm^3^ μmol^-1^ yr^-1^		
*k*_*nhfo*_ (*)	NH_4_ oxidation by reactive FeOOH	9.45 x 10^−4^	cm^3^ μmol^-1^ yr^-1^		
*k*_*nhfx*_ (*)	NH_4_ oxidation by less-reactive FeOOH	9.45 x 10^−5^	cm^3^ μmol^-1^ yr^-1^		
*k*_*fmo*_ (*)	Fe(II) oxidation by reactive MnO_2_	1.5 x 10^−2^	cm^3^ μmol^-1^ yr^-1^	1–3000	[19]
*k*_*fmx*_ (*)	Fe(II) oxidation by less-reactive MnO_2_	1.5 x 10^−3^	cm^3^ μmol^-1^ yr^-1^		
*k*_*smo*_ (*)	S(-II) oxidation by reactive MnO_2_	1	cm^3^ μmol^-1^ yr^-1^	10–20	[8, 19]
*k*_*smx*_ (*)	S(-II) oxidation by less-reactive MnO_2_	0.1	cm^3^ μmol^-1^ yr^-1^		
*k*_*sfo*_ (*)	S(-II) oxidation by reactive FeOOH	2.5	cm^3^ μmol^-1^ yr^-1^	10−2–10^2^	[8, 19]
*k*_*sfx*_ (*)	S(-II) oxidation by less-reactive FeOOH	0.25	cm^3^ μmol^-1^ yr^-1^		
*k*_*chso*_ (*)	CH_4_ oxidation by sulfate	0.5	cm^3^ μmol^-1^ yr^-1^	10	[8]
*k*_*moN*_ (*)	Mn(II) oxidation by nitrate	1 x 10^3^	cm^3^ μmol^-1^ yr^-1^		
*k*_*vivpre*_ (**)	Vivianite precipitation	2.72 x 10^−5^	μmol g^-1^ yr^-1^	10^−4^–1.7 x 10^−3^	[19, 20]
*k*_*vivdis*_ (**)	Vivianite dissolution	1	yr^-1^	1	[19]
*k*_*EqViv*_ (**)		3 x 10^−20^		3 x 10^−20^	[19]
*k*_*sviv*_ (**)	Vivianite dissolution by S(-II)	10	cm^3^ μmol^-1^ yr^-1^	10–100	[19]
*k*_*IronSulfidePre*_ (**)	FeS precipitation	0.1	μmol g^-1^ yr^-1^	0.1–100	[19]
*k*_*IronSulfideDis*_ (**)	FeS dissolution	2 x 10^−3^	yr^-1^	1 x 10^−3^	[19]
*k*_*EqIronsulfide*_ (**)		6.3096			[19]
*k*_*pyrpre*_ (**)	FeS_2_ precipitation	4	cm^3^ μmol^-1^ yr^-1^	10−3–10^3^	[19, 20]
*k*_*MnCarbonatePre*_ (*)	MnCO_3_ precipitation	0.01064	μmol g^-1^ yr^-1^	10–10000	[19]
$k_{Eq{HCO}_{3}{CO}_{3}}$ (**)		2375 x 10^−8^			
*k*_*EqMnCarbonate*_ (**)		0.022		0.022	[19]
H^+^ (pH) (**)		10^−7^			
L (*)	Rate limiting constant	10^−8^			

## Results and discussion

### Model calibration

The values of the model parameters (Table 3) were initially set either based on literature values or field observations. Boundary conditions were subsequently modified during the calibration process, using a manual trial-and-error approach (Table 1). Further, reaction rates taken from similar model studies by Van Cappellen and Wang [8], Dittrich, Wehrli [10] and Katsev and Dittrich [19] were used as initial rates. First, by using these initial rates, relative fractions of reactive to non-reactive pools of OM, Fe, and Mn were determined by visually comparing the modeled and measured porewater and sediment profiles. Then, process rates were calibrated to further improve, reproduce and match the measured porewater concentration profiles and sediment concentrations without using numerical fitting procedures (see asterisks Table 3).

The sensitivity of the model results to individual process rates was assessed by step-wise increasing/decreasing (±1%) the respective rate and comparing the resulting AHM to that from the baseline run. Sensitivities in the following are expressed as relative change in the model output divided by relative change of the model parameter. The sensitivity of O_2_ consumption and F_red_ production to most of the process rates was low (below <0.01), but adjusting these rates was required for reproducing the observed concentrations of individual species e.g Fe(II) oxidation rate for total solid Fe or the Fe(II) profile. The highest sensitivities of AHM were found for the deposition rate of OM_1_ (1.04) and the rate of the main carbon mineralization pathway methanogenesis (*k*_*deg*,*CH4*_) with 0.25. Oxic degradation (*k*_*deg*,*O2*_) showed a surprisingly low sensitivity of **~**0.20. The other main carbon mineralization pathways showed lower sensitivities with denitrification (*k*_*deg*,*NO3*_) **~**0.04, FeOOH reduction (*k*_*deg*,*FeOOH*_) <0.01, MnO_2_ reduction (*k*_*deg*,*MnO2*_) <0.01 and sulfate reduction (*k*_*deg*,*SO4*_) <0.01.

Based on the fluxes across the sediment-water interface, the total mass within the sediment and the amounts leaving the modeled sediment depth range at the bottom, the total mass balance of each element species was calculated. Mass balances of all species were preserved. Carbon and nitrogen mass balances are shown in S1 Fig.

**Fig 2:**
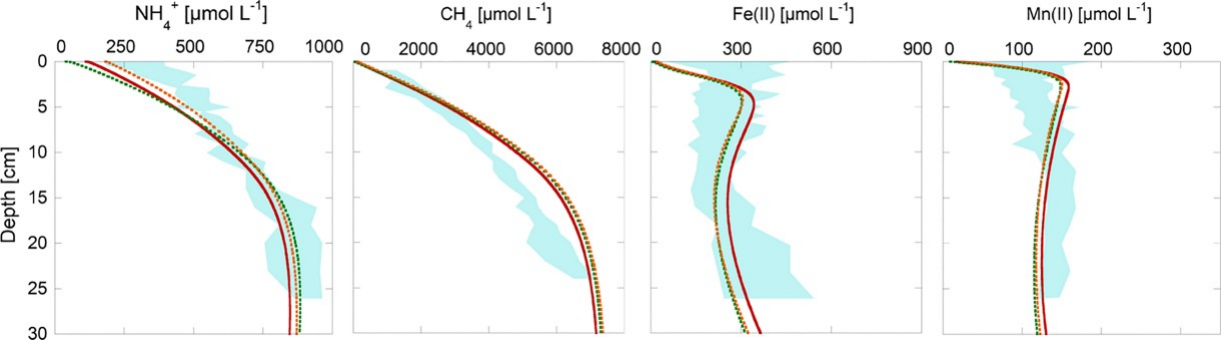
All measured porewater concentrations for the years 2014 and 2015 and modeled porewater concentrations of 2015. The blue shaded area indicates the seasonal porewater variations encountered in the field campaign. The red line is the modeled porewater concentration using average input parameters. The dotted lines represent seasonal modeled concentrations during the middle of the year (mid-June, orange) and the end of the year (mid-December, green).

The first goal of the calibration was to reproduce observed porewater profiles of the most important species contributing to F_red_ and their respective fluxes from the sediment to the water column. The measured and modeled results for NH_4_^+^, CH_4,_ Fe(II) and Mn(II) are shown in Fig 2.

### Porewater profiles

In agreement with sediment porewater measurements [2], modeled porewater concentrations of NH_4_^+^ and CH_4_ increased with increasing sediment depth. This was mainly a result of the aerobic mineralization at the top of the sediment and the dominating process of methanogenesis in the deeper parts of the sediment. While modeled and measured CH_4_ concentrations were similar in the top few of centimeters, the deviation increased with increasing sediment depth. Reproduction of the observed shape of the CH_4_ profiles required an additional CH_4_ sink below the oxic zone or changes of the C/N ratio in the OM pool. Several anaerobic oxidation processes of CH_4_ such as oxidation by ferric iron oxides [30], Mn(IV)-oxides [31] or humic compounds [32] might play a role here but are not implemented in the reaction network.

Modeled and measured porewater profiles of Fe(II) and Mn(II) were also in reasonable agreement, despite their complex shapes due to various precipitation and dissolution reactions. The Fe(II) peak is caused by the initial reduction of iron (hydr-)oxides. The resulting ferrous iron diffuses towards the sediment surface where it is rapidly re-oxidized to various forms of ferric (hydr-)oxides creating an iron redox cycle. Although the major reactions for Fe(III) and Mn(IV) precipitation and reductive dissolution were included in the model, we acknowledge that without simultaneous pH calculations their significance remains limited. However, as Fe(II) and Mn(II) concentrations and hence fluxes only contributed a very small fraction to the total F_red_ [3, 7], their values had a negligible effect on the overall picture.

**Fig 3:**
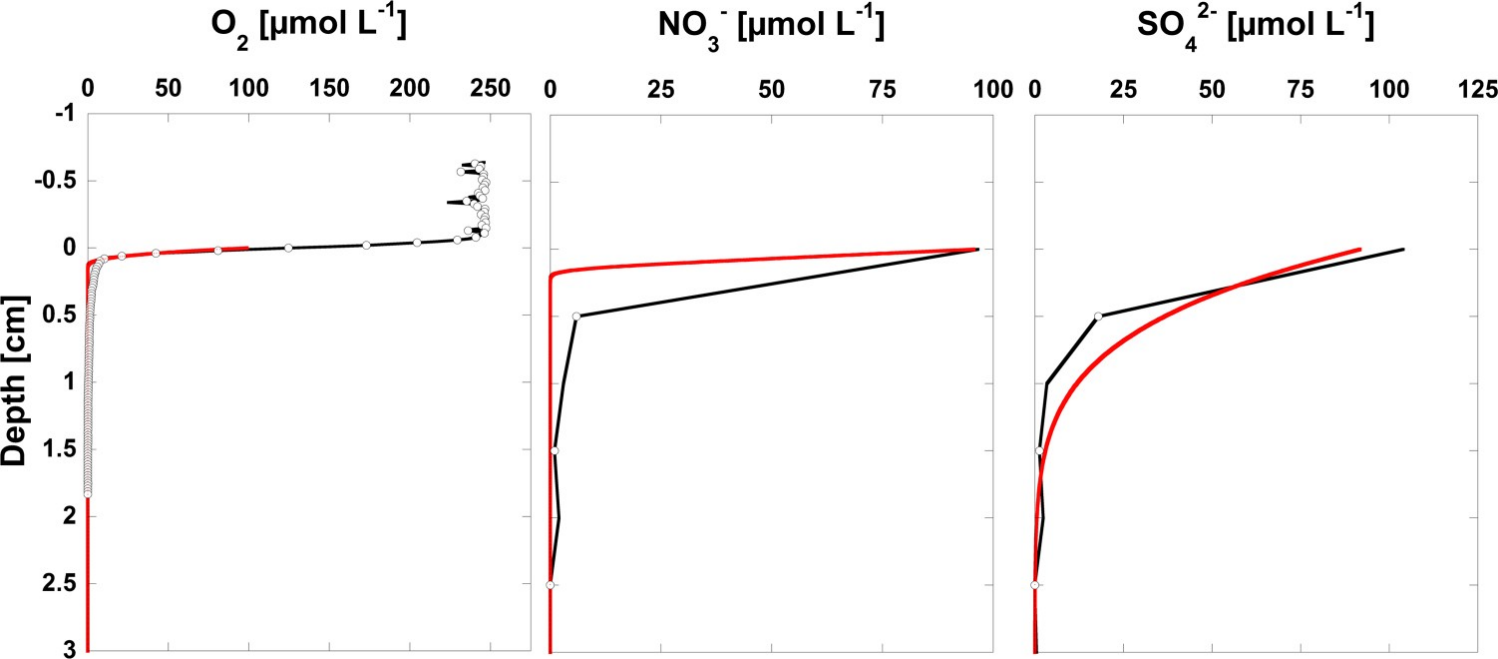
Modeled (red line) and measured (black line) porewater concentrations of the terminal electron acceptors O_2_, NO_3_^-^ and SO_4_^2-^. Direct O_2_ measurements were taken only once during winter overturn.

Measured and modeled sediment porewater concentrations of O_2_, NO_3_^-^ and SO_4_^2-^ are shown in Fig 3. O_2_ measurements were taken during winter overturn with highest O_2_ concentrations of around 245 μmol L^-1^ in the sediment overlying water and decreasing to zero within 2 mm below the diffusive boundary layer. Similar rapid drops in porewater concentrations were observed for NO_3_^-^ and SO_4_^2-^, where porewater concentrations decrease to zero within 5 mm and 15 mm, respectively.

### Fluxes of reduced substances (F_red_)

The porewater fluxes were estimated from measured porewater concentration profiles using Fick’s first law of diffusion. The fluxes where then expressed as O_2_ equivalents according to their oxidation stoichiometry and summed up to F_red_ [3]. The F_red_ flux resulting from the sediment model for 2015 was 0.36 gO_2_ m^-2^ d^-1^. The modeled value is close to the value determined with the steady-state 1-D model by Epping and Helder [22] of 0.49±0.09 gO_2_ m^-2^ d^-1^ in 2015 [2]. Likewise modeled and measured CH_4_ fluxes were in perfect agreement with 0.22 and 0.25±0.06 gO_2_ m^-2^ d^-1^ respectively. Similarly, modeled and measured NH_4_ fluxes were in good agreement with 0.13 and 0.18±0.07 gO_2_ m^-2^ d^-1^ respectively. Modeled CH_4_ and NH_4_^+^ fluxes contributed 61.1% and 34.7% to F_red_, respectively, while measured CH_4_ and NH_4_^+^ fluxes contributed 55.1±5.6% and 41.7±5.7% to F_red_. Modeled and measured Fe(II) and Mn(II) fluxes were 2x10^-3^ and 3±1x10^-3^ gO_2_ m^-2^ d^-1^ and 6x10^-3^ and 1±1x10^-3^ gO_2_ m^-2^ d^-1^, respectively. Modeled Mn(II) and Fe(II) fluxes contributed 1.7% and 0.5% to F_red,_ observed values were 0.7±0.5% and 1.8±0.3%, respectively.

Modeled sulfide (S-II) fluxes accounted for 3.3% of F_red_, although dissolved S(-II) has not been detected in the field.

### Sediment TOC

**Fig 4:**
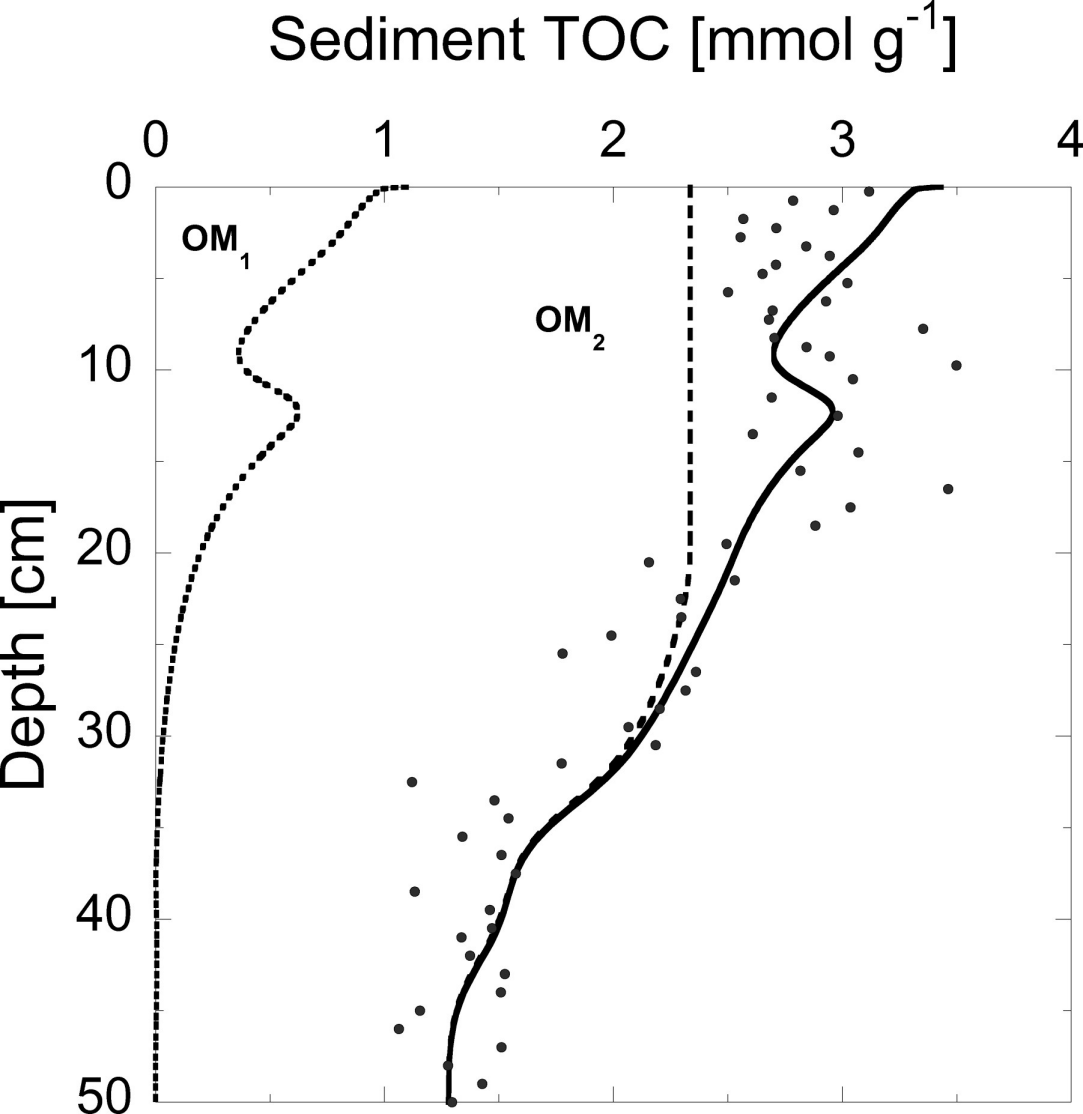
Vertical profiles of measured (dots) and simulated (line) TOC concentrations (2015). Simulated concentrations are further split up into reactive OM_1_ (dotted) and non-reactive OM_2_ (dashed) TOC pools. The “bump” in the modeled TOC concentration between 10 and 15 cm depth is caused by the onset of artificial aeration in 1982.

To reproduce the observed vertical profiles of TOC in the sediment, we calibrated the ratio between reactive and non-reactive OM. A good agreement between simulated and observed TOC was reached under the assumption that two thirds of the total TOC gross sedimentation are reactive with an absolute error of ~ 7.5% between the measured and modeled TOC values (Fig 4). This assumption seems reasonable for a highly productive lake such as Lake Baldegg. A large fraction of the reactive TOC pool (OM_1_) was mineralized directly at the sediment surface, and decreased by about 50% within the top 10 centimeters of the sediment. Deeper in the sediment, the largest TOC pool consisted of non-reactive TOC, and no reactive TOC was left below 33 cm.

### Simulated scenarios

The calibrated model is an excellent tool for assessing the future development of the O_2_ consumption rate in the lake applying different management strategies. The transient boundary conditions of the model were adjusted to simulate four different scenarios up to the year 2050. Each setup started with the same initial conditions in 1850, and boundary conditions were only changed after 2018 to account for the four abovementioned scenarios: “Status Quo” (SQ), Mesotrophic production (M), “Oligotrophic production” (O) and “No artificial aeration” (NoAa). The underlying premise for all settings (except NoAa) is that artificial aeration remains active to supply sufficient hypolimnetic O_2_. Furthermore, all scenarios were modeled twice, once with no seasonal variations and once with simplified seasonal variations of the changing input parameters described in Table 1.

### SQ scenario (Status quo continued)

**Fig 5:**
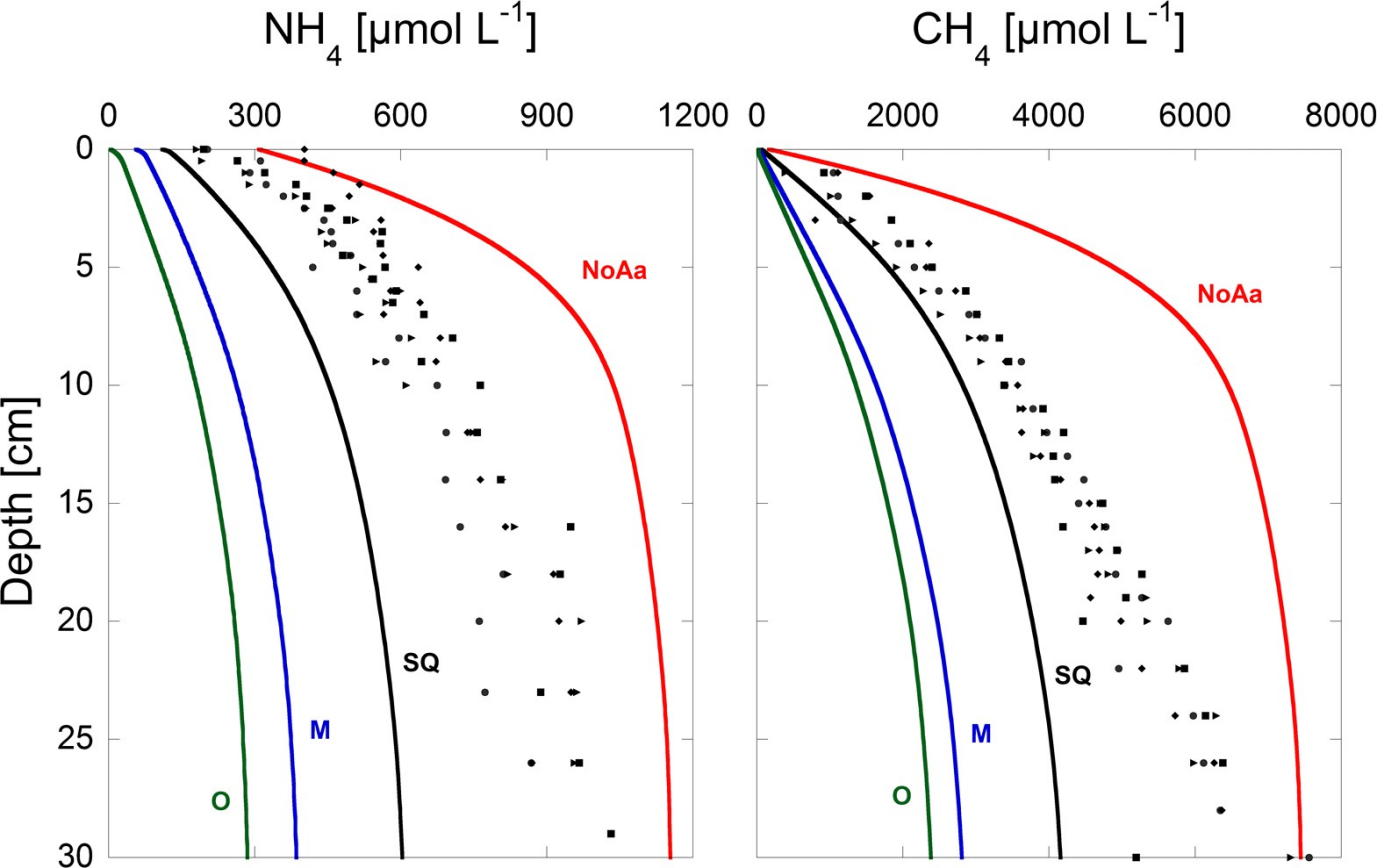
Modeled CH_4_ and NH_4_ porewater concentrations for the year 2050. The colored lines represent results for the different scenarios: red (no artificial aeration), black (status quo), blue (mesotrophic) and green (oligotrophic). In comparison, the porewater concentrations measured in the year 2015 are plotted with black dots.

The modeled concentration profiles of NH_4_^+^ and CH_4_ for the year 2050 in the various scenarios are shown in Fig 5.

**Fig 6:**
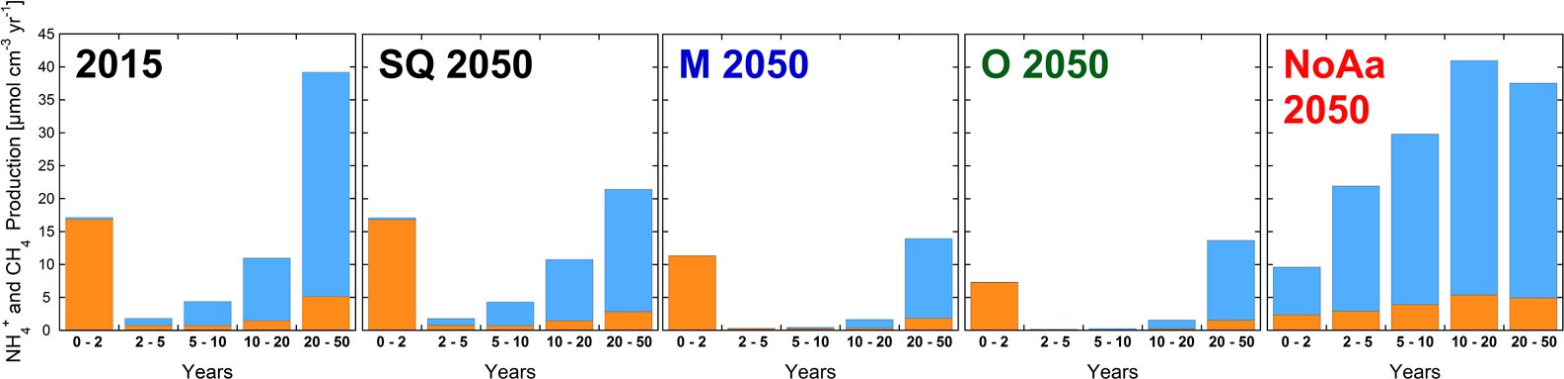
NH_4_^+^ (orange) and CH_4_ (blue) production rates (in μmol cm^-3^ yr^-1^) at different sediment depths (split up in layers corresponding to different ages since sediment deposition) for the year 2015, and for the year 2050 in the SQ, M, O and the NoAa scenario. Note that as the scenarios have different sedimentation rates, the age of the layers at a certain depth differs between the scenarios.

In the SQ scenario, NH_4_^+^ fluxes are projected to decrease by 8.6% from 2015 to 2050. In contrast, CH_4_ fluxes (37%) decline more rapidly until 2050. NH_4_^+^ is produced during each mineralization step of organic matter, while CH_4_ is solely the result of methanogenesis, which occurs deeper in the sediment and is fueled by the buried reactive TOC (see Fig 6).

In 2015, the methanogenesis in sediments older than 20 years contributed ~20% of NH_4_^+^ production but ~70% of the CH_4_ production. In contrast, 68% of the NH_4_^+^ released from the sediments originate from OM deposited during the last two years and, therefore, its contribution indirectly reflects the current primary production.

**Fig 7:**
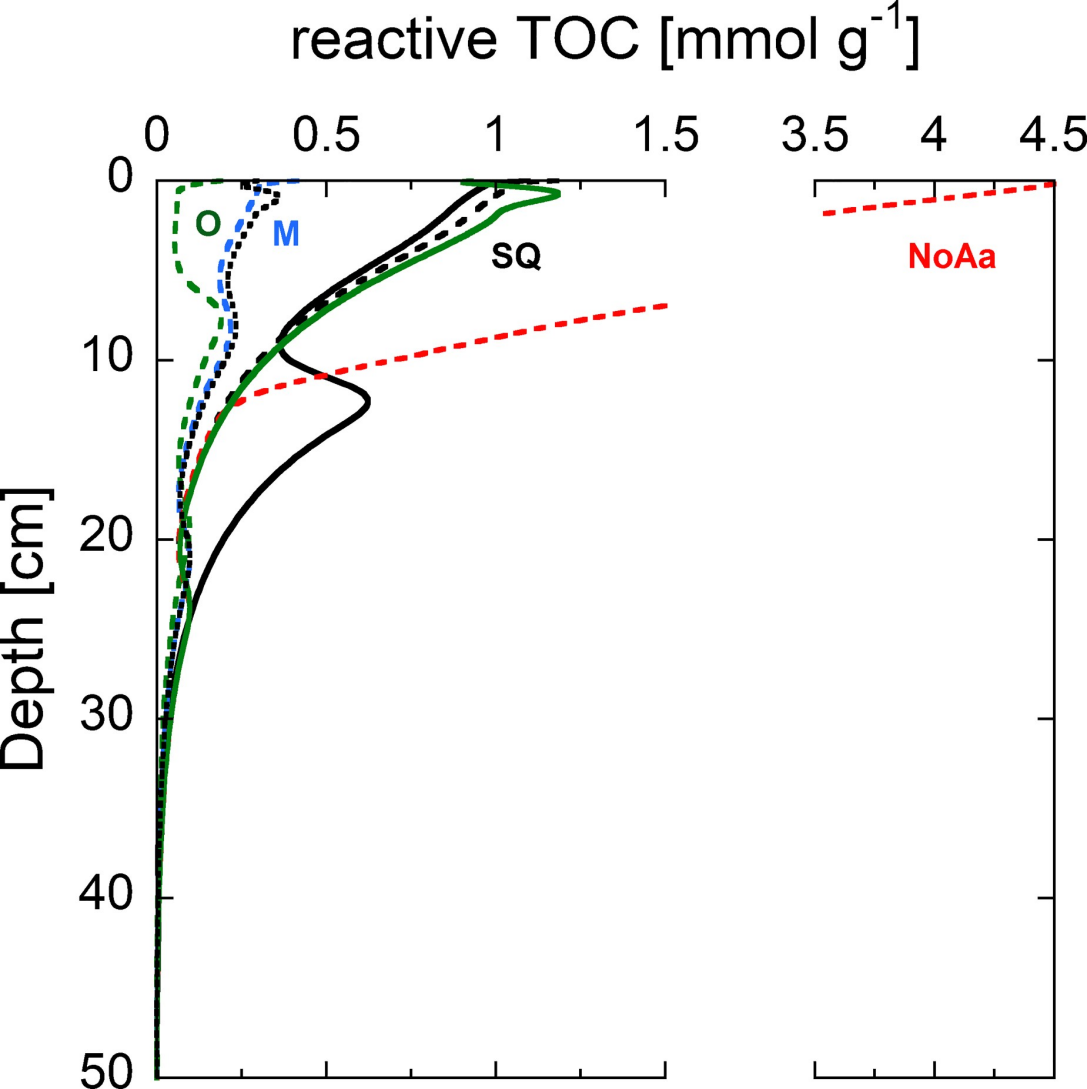
Modeled concentrations of reactive TOC in the different scenarios in 2050 (dashed lines). The large accumulation of reactive TOC in the NoAa scenario (red dashed) is due to anoxic conditions. The solid black line represents modeled reactive TOC concentrations in 2015 for comparison.

By 2050, the contribution to NH_4_^+^ and CH_4_ production by sediments older than 20 years will decrease by ~55%, whereas almost no change in sediments younger than 20 years occurs (see Fig 6). This suggests that the upper sediment strata will remain in quasi steady state with the current TOC gross sedimentation and O_2_ concentration, while the stock of buried reactive TOC from the era of hypertrophy and anoxia decreases over time (see Fig 7). The SQ assumption shows that the present share of NH_4_^+^ and CH_4_ production from sediments older than 20 years is 53% and will decrease to 39% by the year 2050 (Fig 6) These results are in agreement with previous studies. Matzinger, Müller [7] calculated that in Türlersee and Pfäffikersee, deep buried organic rich sediments (>20 years) contributed up to 29% of the CH_4_ and NH_4_^+^ fluxes from the sediment.

**Fig 8:**
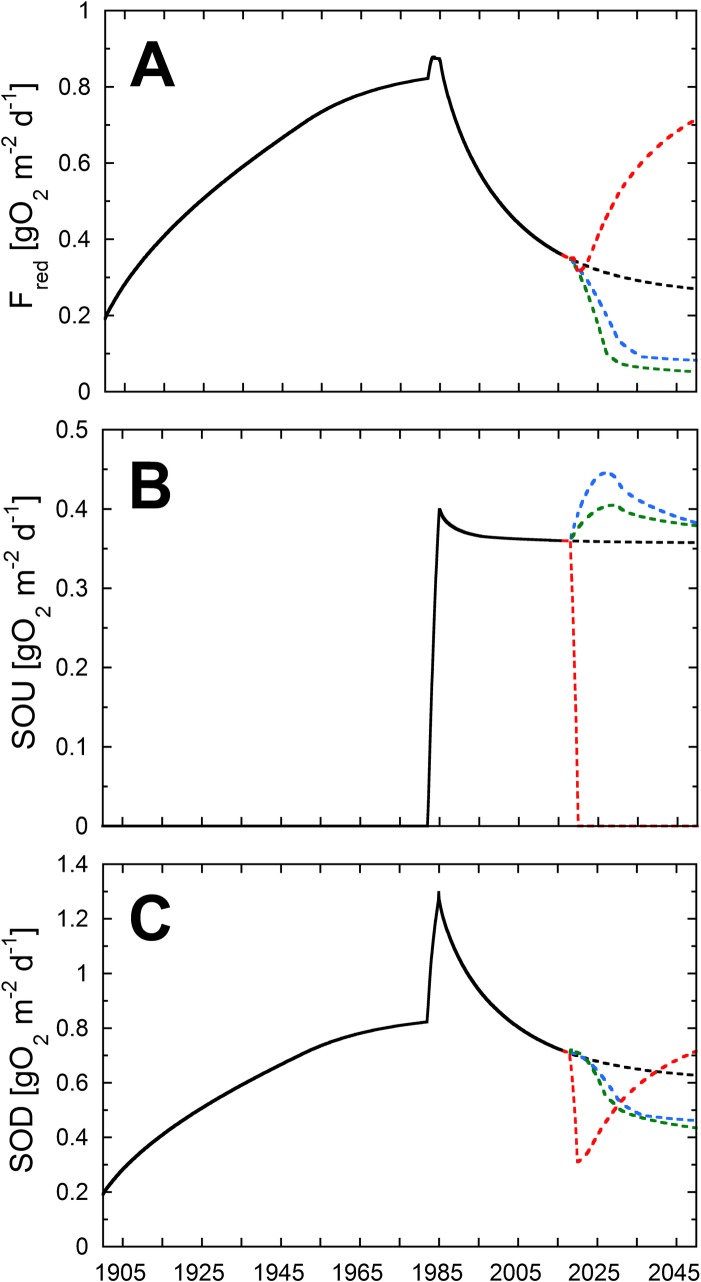
**(C) O_2_ consumption by the two O_2_ sinks F_red_ (A) and SOU (B) in gO_2_ m^-2^ d^-1^.** The solid black line shows the modeled values until 2018. The dashed lines indicate the different modeling scenarios: SQ (black), M (blue), O (green) and NoAa (red).

Total F_red_ will decrease by 23% from 0.35 gO_2_ m^-2^ d^-1^ (2015) to 0.27 gO_2_ m^-2^ d^-1^ (2050) until 2050 (Fig 8A), while SOU remains unchanged at 0.36 gO_2_ m^-2^ d^-1^ (Fig 8B). Hence, SOD will decrease only by 13% until 2050 (see Fig 8C), largely as a consequence of the diminishing pool of reactive TOC.

### Reoligotrophication scenarios

The mesotrophic (M) and oligotrophic (O) scenarios consider the potential decrease of DO consumption if further measures for reoligotrophication are implemented in the lake and its catchment. We assumed the TOC flux to be reduced to 60 gC m^-2^ yr^-1^ for the M scenario and to 36 gC m^-2^ yr^-1^ for the O scenario. Simultaneously, average O_2_ concentrations are increased from 3.2 mgO_2_ L^-1^ (in SQ) to 4.8 mgO_2_ L^-1^ (in M) and to 6.4 mgO_2_ L^-1^ (in O) over a 12-year period from 2018 to 2030. These changes lead to decreased NH_4_^+^ and CH_4_ production rates in the sediments (Fig 6). Sediment CH_4_ production decreases by ~58% for both the M and O scenarios compared to the SQ scenario. Interestingly, the eventual CH_4_ production is not smaller for the O than for the M scenario, probably because already in the mesotrophic state, TEA concentrations are sufficient to mineralize enough TOC and thus minimize the supply for methanogenesis. In contrast to the present state and the SQ scenario, mineralization of reactive TOC is largely completed within the top few centimeters and only a small fraction of reactive TOC is available for methanogenesis deeper in the sediment (see Figs 6 and 7). In both reoligotrophication scenarios, the main fraction of CH_4_ (88%) originates from sediments older than 20 years. NH_4_^+^ production decreases by 38% in the M scenario and by 59% in the O scenario. In contrast to CH_4_ production, the largest decrease in NH_4_^+^ production occurs in the top two centimeters of the sediment and reflects the decreased deposition and increased initial mineralization of OM (see Fig 6).

The NH_4_^+^ flux decreases by 43% in the M scenario and by 62% in the O scenario. The CH_4_ flux decreases to zero in both scenarios, as all upward diffusing CH_4_ is oxidized within a small oxic layer of the sediment. Consequently, the relative O_2_ consumption by CH_4_ oxidation increases from 20% in the SQ to 50% in M scenario to 70% in the O scenario. Although a higher O_2_ concentration was chosen in the O scenario, the elevated O_2_ concentration does not increase the SOU rate (see Fig 8B). In fact, SOU rates in the M and O scenarios converge with growing influence of CH_4_ oxidation as a major O_2_ sink. Further, the O_2_ penetration depth (O_2_ concentration >0.1 μmol L^-1^) increases from 0.95 mm in the SQ scenario to 4 mm in the O scenario.

While SOU is predicted to increase from 0.36 gO_2_ m^-2^ d^-1^ in 2015 to 0.38 gO_2_ m^-2^ d^-1^ by 2050, F_red_ decreases from 0.36 gO_2_ m^-2^ d^-1^ to only 0.08 gO_2_ m^-2^ d^-1^ in the M scenario, to 0.05 gO_2_ m^-2^ d^-1^ in the O scenario and only contributes 12% and 18% to SOD, respectively (see Fig 8A and 8B). Hence, in the reoligotrophication scenarios SOD decreases by over 36% and is mainly controlled by SOU and, therefore, by the lake’s current primary production.

### No artificial aeration scenario (NoAa)

Without measures to reduce primary production and without artificial hypolimnetic aeration promoting aerobic mineralization of TOC, large amounts of reactive TOC would be buried in the sediments of Lake Baldegg (see Fig 7). Under the resulting anoxic conditions bioturbation rapidly decreases (see Table 1). The high deposition of reactive TOC (see Fig 6) increases the production of NH_4_^+^ and CH_4_ in all sediment depths (see Fig 5). As a result, methanogenesis dominates TOC mineralization in 2050 with up to 93% in contrast to only about 30% in 2015. Furthermore, fluxes of NH_4_^+^ and CH_4_ increase by 23% and 191%. In total, F_red_ doubles from 0.36 gO_2_ m^-2^ d^-1^ to 0.72 gO_2_ m^-2^ d^-1^ by 2050 (see Fig 8A). The projected increase of the NH_4_^+^ flux from the sediments is rather modest due to the increasing concentration of NH_4_^+^ in the bottom water (300 μmol L^-1^). The boundary condition for CH_4_ was set to 100 μmol L^-1^, however, we acknowledge that much higher concentrations might be possible. In Lake Rotsee, a highly productive and eutrophic lake, CH_4_ concentrations reach the gas saturation concentration directly below the sediment surface during summer stagnation, and CH_4_ is therefore additionally lost to the hypolimnion by bubble formation [33]. SOU obviously is zero during permanent anoxia (see Fig 8B), and SOD consists only of F_red_ (0.72 gO_2_ m^-2^ d^-1^). However, once aeration is restarted, a large spike in SOD, similar to the spike following the begin of aeration in 1982, is expected to occur. The large TOC pool buried during the anoxic phase (2020–2050) would cause high F_red_ values for decades (similar to the reactive TOC pool buried during the anoxic phase between 1890 to 1982). This would impede any efforts of ensuing re-oligotrophication measures and artificial aeration.

### Seasonal modeling

**Fig 9:**
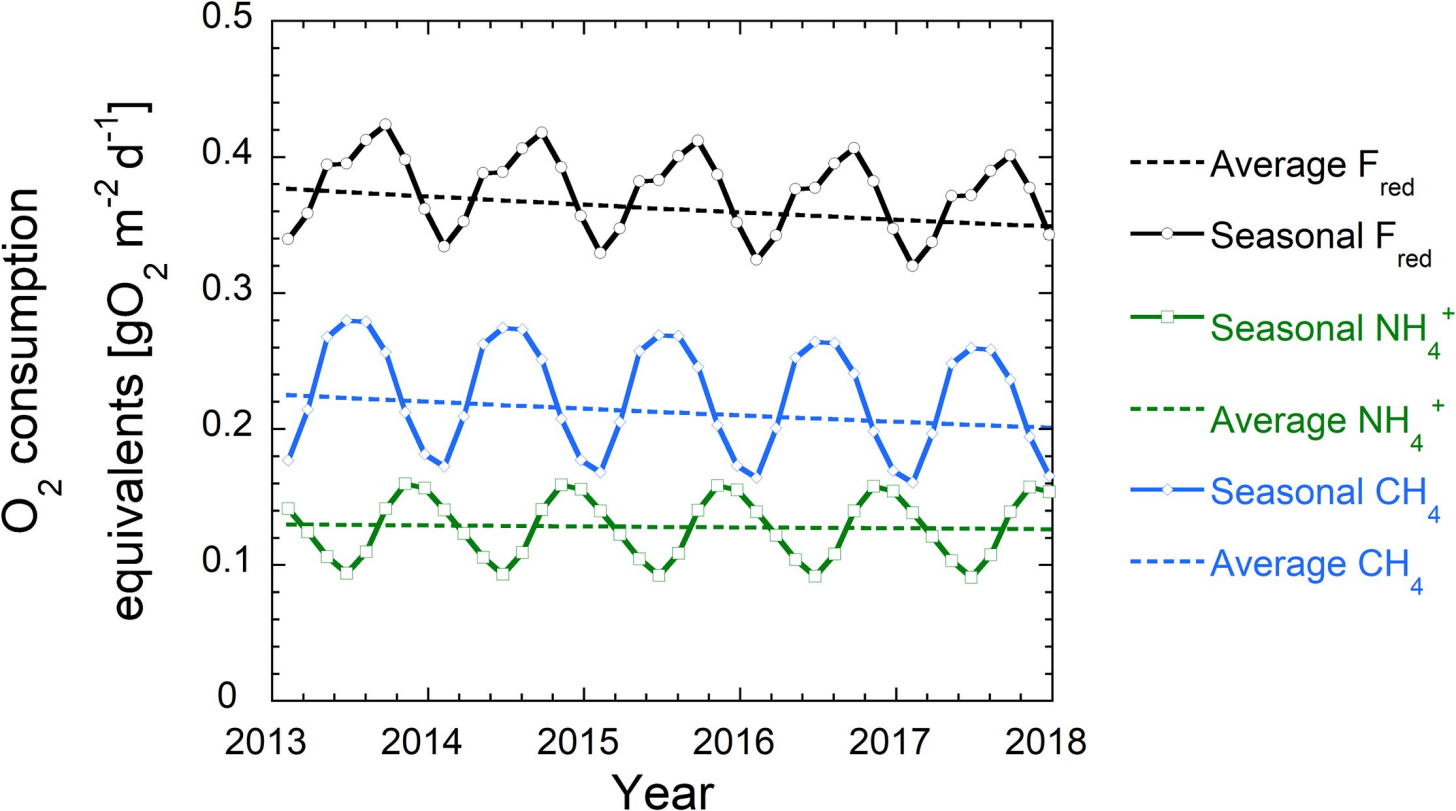
Modeled seasonal and average fluxes of CH_4_ and NH_4_^+^ expressed in O_2_ consumption equivalents (F_red_). While CH_4_ fluxes (blue) decreased over time as less buried TOC was available for methanogenesis, NH_4_^+^ fluxes (green) remained almost constant as they were dominated by the initial steps of anaerobic degradation. Seasonal fluxes of CH_4_ and NH_4_^+^ oscillated due to the seasonally changing prevalence of aerobic and anaerobic degradation.

In the seasonal models, concentrations and fluxes of TOC, NH_4_^+^_,_ CH_4_, Mn(II) and Fe(II) increased during summer stratification, while concentrations of O_2_, NO_3_^-^ and SO_4_^2-^ decreased (see Fig 1). Consequently, during the time of the highest TOC deposition rate, the lowest concentrations of TEAs occurred. This resulted in the highest fluxes of F_red_ at the end of summer stratification (Fig 9) as increasingly more material is degraded anaerobically at that time. Lowest F_red_ values occurred during winter overturn, where highest O_2_ concentrations and lowest TOC gross sedimentation prevailed. Hence, during winter overturn the deposited TOC is increasingly exposed to and mineralized by O_2_ thereby diminishing the storage of OM in the sediment.

**Fig 10:**
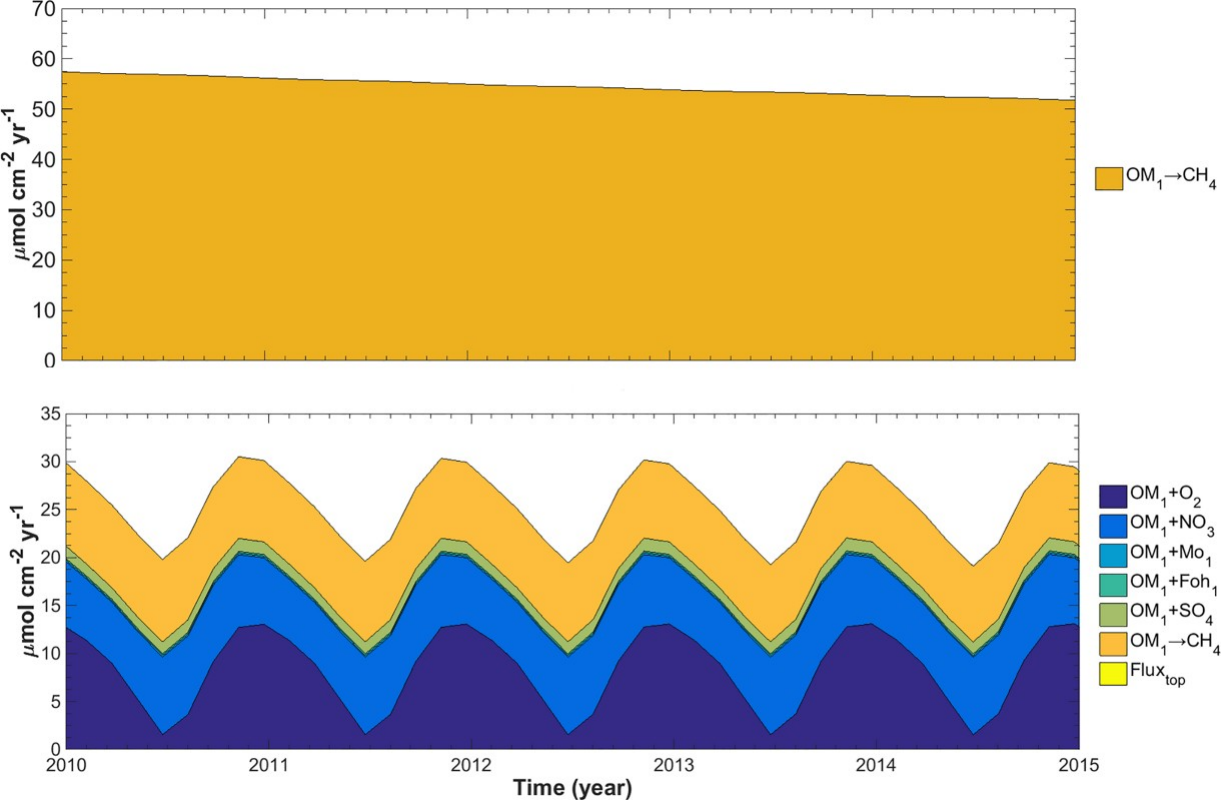
Production of CH_4_ (top) and NH_4_^+^ (bottom) for the seasonal SQ model from 2010 to 2015. The colors indicate the pathway of production.

Since most of the CH_4_ was produced in the deeper parts of the sediment, its rate was independent of seasonal variations (Fig 10). It decreased only when old deposits of reactive TOC were exhausted. However, seasonal variations of CH_4_ fluxes are induced by varying O_2_ penetration depths (see Fig 8). In contrast to CH_4_, only ~30% of the NH_4_^+^ is produced during methanogenesis (Fig 10). Aerobic mineralization of OM (≤ 37%) and dissimilatory reduction of nitrate to ammonium (≤ 27%) together produce up to 64% of NH_4_^+^. As O_2_ concentrations in the hypolimnion decrease during stratification, the production of NH_4_^+^ via aerobic mineralization diminishes likewise. The seasonal impact of other TEAs such as NO_3_^-^, Fe(III)-(hydr)oxides, Mn(IV)-oxides and SO_4_^2-^ on the overall NH_4_^+^ production is negligible. Conversely, the fraction of aerobically degraded TOC increases at the end of each year. In contrast to CH_4_, both the production and fluxes of NH_4_^+^ show distinct seasonal variations (Fig 10).

**Fig 11:**
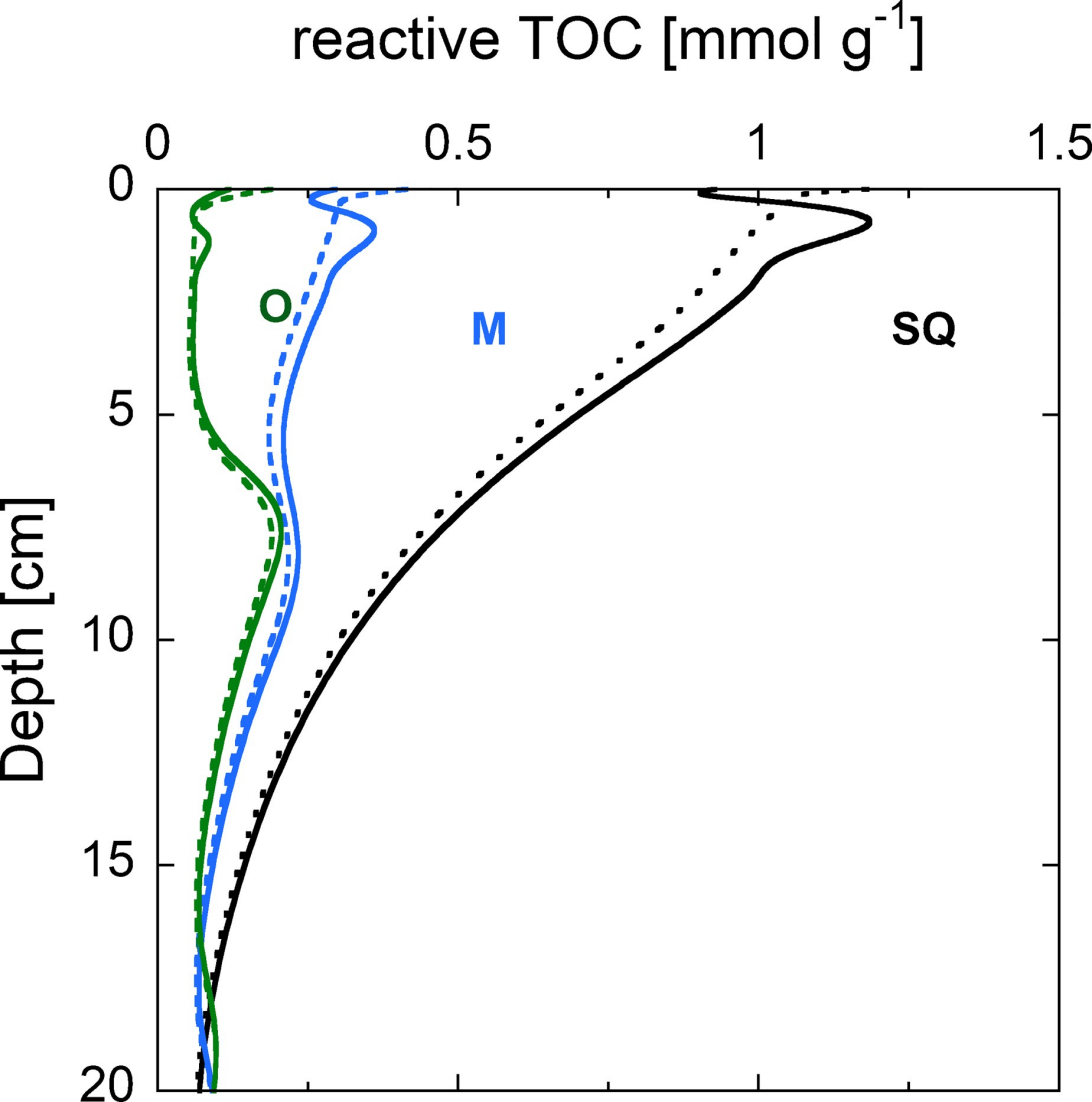
Content of reactive TOC in the sediments in 2050 modeled with average boundary conditions (dotted) and seasonally varying conditions (solid lines) for three different scenarios: SQ (black), M (blue) and O (green) in the middle of the year.

Seasonal variations of fluxes of reduced substances were observed in several lakes [2, 7, 34]. The coincidence of highest TOC gross accumulation and lowest TEA concentrations in the middle of any given year leads to higher accumulation rates of reactive TOC and thus to increased burial of reactive TOC in the sediments (see Fig 11) [12]. However, this effect is only apparent in the seasonal models.

As more reactive TOC is buried, slightly higher fluxes of F_red_ are encountered in the seasonal models compared to non-seasonal models (see Fig 9). The average F_red_ modeled for the year 2015 is 0.36 gO_2_ m^-2^ d^-1^ in the SQ scenario, and 0.38±0.03 gO_2_ m^-2^ d^-1^ for the seasonal model. Caused by the seasonal changes in O_2_ concentrations and aerobic degradation, SOU and consequently SOD show higher variability. Averaged SOD values between 2030 and 2050 in the non-seasonal SQ model are 0.64±0.01 gO_2_ m^-2^ d^-1^, comparable to the 0.63±0.17 gO_2_ m^-2^ d^-1^ of the seasonal SQ model.

In conclusion, simplified seasonal variations of the upper boundary condition cause seasonal variations in the NH_4_^+^ production, slightly higher accumulation rates of reactive TOC, variations in F_red_, increased SOU and consequently, slightly higher O_2_ depletion rates. Although the overall impact of seasonal boundary variation is negligible, they are important to explain observed porewater profiles of species such as NH_4_^+^, Fe(II) and Mn(II), which have been shown to vary seasonally, especially in the top five centimeters of the sediments (see Fig 2)[2].

## Conclusion

A multicomponent early diagenesis model with transient boundary conditions was developed and calibrated using historic observations, monitoring data and recent state of the art porewater data from the eutrophic and artificially aerated Lake Baldegg. The model was applied to simulate four different management scenarios, including a continuation of the status quo, two re-oligotrophication scenarios, and a scenario where lake aeration is stopped. Simulations with seasonal boundary conditions yielded similar O_2_ depletion rates as the non-seasonal model, implying that it is not necessary to consider seasonality and thus increase computation time for long-term projections of sediment oxygen demand.

All modeled scenarios show the direct impact of the TOC gross sedimentation rate and the bottom water O_2_ concentration on the hypolimnetic O_2_ depletion rates. While the upper boundary O_2_ concentration is doubled between the scenarios (SQ to O), the sediment oxygen uptake (SOU) differs only slightly. However, less reactive TOC is buried which significantly decreases F_red_. Although older sediments (deposited during the hypertrophic and anoxic period) dominate F_red_ due to methanogenesis, this “sediment memory effect” [7] or “legacy carbon effect” [6] is overcome once primary production decreases. This is shown in both reoligotrophication scenarios, where SOD is mostly controlled by SOU. Interestingly, already a change to mesotrophic production yields a SOD rate comparable to a low productive state. Hence, with an adequate O_2_ concentration, a return to mesotrophic productivity could be sufficient for a long-term decline of SOD. In contrast, a termination of the aeration program without measures to decrease TOC gross sedimentation would yield long-lasting damaging effects on the O_2_ consumption in the hypolimnion of Lake Baldegg as large amounts of buried reactive TOC would lead to a high production of CH_4_ and NH_4_^+^ and eventually to hypolimnetic conditions similar to the anoxic phase between 1885 and 1982.

## Supporting information

S1 Fig Mass balances of carbon (top) and nitrogen (bottom). The individual fluxes into the sediment are calculated through their respective upper boundary conditions. The fluxes out of the sediment at bottom are defined as the sediment strata that is moved out of the active modelled area.
(TIF)

S1 Table List of biogeochemical reactions and their respective stoichiometry. (DOCX)

S2 Table Process rates of the reactions in the model. Please note that for each transformed om1, the Redfield ratio equivalent of 106 carbons are liberated. L is used as a rate limitation constant in each reaction.
(DOCX)
