# Supplementary figures and images for: Modeling sediment oxygen demand in a highly productive lake under various trophic scenarios
Source: PLoS One. 2019 Oct 9;14(10):e0222318. doi: 10.1371/journal.pone.0222318 (PMC6784980; doi:10.1371/journal.pone.0222318)

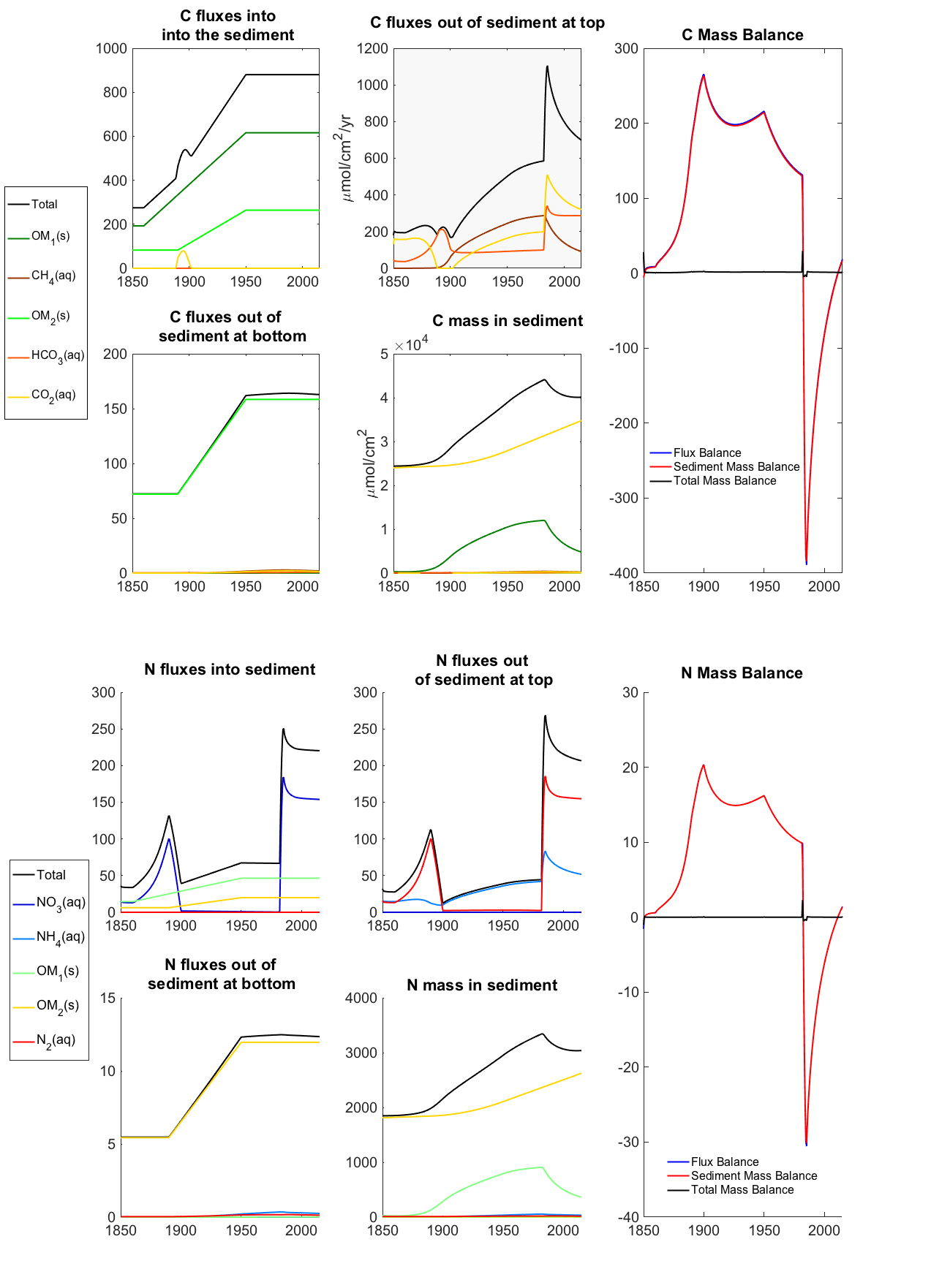

Supplement: S1 Fig — Mass balances of carbon (top) and nitrogen (bottom). The individual fluxes into the sediment are calculated through their respective upper boundary conditions. The fluxes out of the sediment at bottom are defined as the sediment strata that is moved out of the active modelled area. (TIF) [file pone.0222318.s001.tif]
