# Supplementary material for: Modeling sediment oxygen demand in a highly productive lake under various trophic scenarios
Source: PLoS One. 2019 Oct 9;14(10):e0222318. doi: 10.1371/journal.pone.0222318 (PMC6784980; doi:10.1371/journal.pone.0222318)
Supplement: S1 Table — (DOCX) [file pone.0222318.s002.docx]

S1 Table: List of biogeochemical reactions and their respective stoichiometry.

| Rates | Rkn 1 | Rkn 2 | Rkn 3 | Rkn 4 | Rkn 5 | Rkn 6 | Rkn 7 | Rkn 8 | Rkn 9 | Rkn 10 | Rkn 11 | Rkn 12 | Rkn 13 | Rkn 14 | Rkn 15 |
| --- | --- | --- | --- | --- | --- | --- | --- | --- | --- | --- | --- | --- | --- | --- | --- |
| Products | (cy1-2*cz1)*hco3 + (cx1-cy1+2*cz1)*co_2 + (cx1-cy1+1.5*cz1)*h2o + cz1*hpo4 + cy1*nh4 + cs1*s2; | (0.8*cx1+cy1-2*cz1)*hco_3 + (0.2*cx1-cy1+2*cz1)*co_2 +  (0.6*cx1-cy1+2*cz1)*h2o + 0.4*cx1*n2 + cz1*hpo4 + cy1*nh4 + cs1*s2 | (4*cx1+cy1-2*cz1)*hco_3 + 2*cx1*mn + cz1*hpo4 + cy1*nh4 + cs1*s2 | (3*cx1-cy1+2*cz1)*h2o + (8*cx1+cy1-2*cz1)*hco_3 + 4*cx1*fe + cz1*hpo4 + cy1*nh4 + cs1*s2 | (cx1+cy1-2*cz1)*hco_3 + 0.5*cx1*s2 + cz1*hpo4 + cy1*nh4 + cs1*s2 | (0.5*cx1-cy1+2*cz1)*co_2 + (cy1-2*cz1)*hco_3 + 0.5*cx1*ch4 +  cz1*hpo4 + cy1*nh4 + cs1*s2 | no3 + 2*co_2 + 3*h2o | 2*mo_2 + 2*co_2 + 2*h2o | 0.5*foh_1 + 0.5*foh_2 + 2*co_2 | so4 + 2*co_2 + 2*h2o | co_2 + 2*h2o | 3*mn + n2 + 6*h2o | 3*mn + n2 + 6*h2o | foh_1 + foh_2 + mn + 2*co_2 | foh_1 + foh_2 + mn + 2*co_2 |
| Reactants | cx1*om1+(cx1+0.25*cz1)*o2 | cx1*om1 + 0.8*cx1*no3 | cx1*om1 + 2*cx1*mo_1 + (3*cx1+cy1-2*cz1)*co_2 + (cx1+cy1-2*cz1)*h2o | cx1*om1 + 4*cx1*foh_1 +  (7*cx1+cy1-2*cz1)*co_2 | cx1*om1 + 0.5*cx1*so4 + (cy1-2*cz1)*  co_2 + (cy1-2*cz1)*h2o | cx1*om1 + (cy1-2*cz1)*h2o | nh4 + 2*o2 + 2*hco_3 | 2*mn + o2 + 2*hco_3 | fe + 0.25*o2 + 2*hco_3 +0.5*h2o | s2 + 2*o2 + 2*hco_3 | ch4 + 2*o2 | 2*nh4 + 3*mo_1 + 5*h | 2*nh4 + 3*mo_2 + 5*h | 2*fe + mo_1 + 2*hco_3 + 2*h2o | 2*fe + mo_2 + 2*hco_3 + 2*h2o |
| Kinetic Reaction | OM oxidation by O_2_ | OM degradation by nitrate | OM degradation by MnO_2_ | OM degradation by Fe(OH)_3_ | OM degradation by sulfate | Methanogenesis | Nitrification | Mn oxidation by O_2_ | Fe oxidation by O_2_ | H2S oxidation by O_2_ | Methane oxidation by O_2_ | NH_4_ oxidation by 1st MnO_2_ (mo_1) | NH4 oxidation by 2nd MnO2 (mo_2) | Fe oxidation by 1st MnO2 (mo_1) | Fe oxidation by 2nd MnO2 (mo_2) |
| Rates | Rkn 16 | Rkn 17 | Rkn 18 | Rkn 19 | Rkn 20 | Rkn 21 | Rkn 22 | Rkn 23 | Rkn 24 | Rkn 25 | Rkn 26 | Rkn 27 | Rkn 28 | Rkn 29 | Rkn 30 |
| Products | 4*mn + so4 + 6*hco_3 | 4*mn + so4 + 6*hco_3 | 8*fe + so4 + 14*hco_3 + 6*h2o | 8*fe + so4 + 14*hco_3 + 6*h2o | 2*hco_3 + s2 | mo_2 + 0.2*n2 + 1.6*co_2 + 0.8*h2o | 3*fe + 0.5*n2 + 6*h2o | 3*fe + 0.5*n2 + 6*h2o | Vivianite + 2*h + 8*h2o | 3*fe + 2*hpo4 | 3*fs + 2*hpo4 + 4*h | 2*h + fs | fe + s2 | Pyrite + h2 | mo_3 + co_2 + h2o |
| Reactants | s2 + 4*mo_1 + 6*co_2 + 2*h2o | s2 + 4*mo_2 + 6*co_2 + 2*h2o | s2 + 8*foh_1 + 14*co_2 | s2 + 8*foh_2 + 14*co_2 | ch4 + so4 + co_2 | mn + 0.4*no3 + 1.6*hco_3 | nh4 + 3*foh_1 + 5*h | nh4 + 3*foh_2 + 5*h | 3*fe+2*hpo4 | vivianite + 2*h + 8*h2o | 3*s2 + vivianite | s2 + fe | fs + 2*h | fs + s2 | mn + 2 * hco_3 |
| Kinetic Reaction | H2S oxidation by 1st MnO2 (mo_1) | H2S oxidation by 2nd MnO2 (mo_2) | Fe(OH) reduction by H2S | Fe(OH) 2nd pool reduction by H2S | anaerobic methane oxidation | Mn oxidation by nitrate | NH4 oxidation by 1st Fe(OH)3 (foh_1) | NH4 oxidation by 2nd Fe(OH)3 (foh_2) | vivianite precipitation | vivianite dissolution | vivianite dissolution by H2S | Iron sulfide precipitation | iron sulfide dissolution | Pyrite precipitation | Manganese Carbonate precipitation |
