# Supplementary material for: Modeling sediment oxygen demand in a highly productive lake under various trophic scenarios
Source: PLoS One. 2019 Oct 9;14(10):e0222318. doi: 10.1371/journal.pone.0222318 (PMC6784980; doi:10.1371/journal.pone.0222318)
Supplement: S2 Table — Please note that for each transformed om1, the Redfield ratio equivalent of 106 carbons are liberated. L is used as a rate limitation constant in each reaction. (DOCX) [file pone.0222318.s003.docx]

S2 Table. Process rates of the reactions in the model. Please note that for each transformed om_1_, the Redfield ratio equivalent of 106 carbons are liberated. L is used as a rate limitation constant in each reaction.

| Primary reactions : | |
| --- | --- |
| Rkn 1 : $k_{deg,O_{2}}*\frac{{om}_{1}}{{cx}_{1}}*\frac{c_{O_{2}}}{k_{deg,O_{2},inh}+c_{O_{2}}}*\frac{c_{O_{2}}}{(c_{O_{2}}+{om}_{1}*L)}$ | |
| Rkn 2 : $k_{deg,{NO}_{3}}*\frac{{om}_{1}}{{cx}_{1}}*\frac{k_{deg,O_{2},inh}}{k_{deg,O_{2},inh}+c_{O_{2}}}*\frac{c_{{NO}_{3}}}{k_{deg,{NO}_{3},inh}+c_{{NO}_{3}}}*\frac{c_{{NO}_{3}}}{(c_{{NO}_{3}}+{om}_{1}*L)}$ | |
| Rkn 3 : $k_{deg,{MnO}_{2}}*\frac{{om}_{1}}{{cx}_{1}}*\frac{k_{deg,O_{2},inh}}{k_{deg,O_{2},inh}+c_{O_{2}}}*\frac{k_{deg,{NO}_{3},inh}}{k_{deg,{NO}_{3},inh}+c_{{NO}_{3}}}*\frac{c_{mo\_1}}{k_{deg,mo\_1}+c_{mo\_1}}*\frac{c_{mo\_1}}{(c_{mo\_1}+{om}_{1}*L)}$ | |
| Rkn 4 : $k_{deg,FeOOH}*\frac{{om}_{1}}{{cx}_{1}}*\frac{k_{deg,O_{2},inh}}{k_{deg,O_{2},inh}+c_{O_{2}}}*\frac{k_{deg,{NO}_{3},inh}}{k_{deg,{NO}_{3},inh}+c_{{NO}_{3}}}*\frac{k_{deg,mo\_1,inh}}{k_{deg,mo\_1,inh}+c_{mo\_1}}*\frac{c_{foh\_1}}{k_{deg,foh\_1,inh}+c_{foh\_1}}*\frac{c_{foh\_1}}{(c_{foh\_1}+{om}_{1}*L)}$ | |
| Rkn 5 : $k_{deg,{SO}_{4}}*\frac{{om}_{1}}{{cx}_{1}}*\frac{k_{deg,O_{2},inh}}{k_{deg,O_{2},inh}+c_{O_{2}}}*\frac{k_{deg,{NO}_{3},inh}}{k_{deg,{NO}_{3},inh}+c_{{NO}_{3}}}*\frac{k_{deg,mo\_1,inh}}{k_{deg,mo\_1,inh}+c_{mo\_1}}*\frac{k_{deg,foh\_1,inh}}{k_{deg,foh\_1,inh}+c_{foh\_1}}*\frac{c_{{SO}_{4}}}{k_{deg,{SO}_{4}}+c_{{SO}_{4}}}*\frac{c_{{SO}_{4}}}{(c_{{SO}_{4}}+{om}_{1}*L)}$ | |
| Rkn 6 : $k_{deg,{CH}_{4}}*\frac{{om}_{1}}{{cx}_{1}}*\frac{k_{deg,O_{2},inh}}{k_{deg,O_{2},inh}+c_{O_{2}}}*\frac{k_{deg,{NO}_{3},inh}}{k_{deg,{NO}_{3},inh}+c_{{NO}_{3}}}*\frac{k_{deg,mo\_1,inh}}{k_{deg,mo\_1,inh}+c_{mo\_1}}*\frac{k_{deg,foh\_1,inh}}{k_{deg,foh\_1,inh}+c_{foh\_1}}*\frac{k_{deg,{SO}_{4},inh}}{k_{deg,{SO}_{4},inh}+c_{{SO}_{4}}}$ | |
| Secondary reactions : | |
| Rkn 7 : $k_{nhox}*c_{{NH}_{4}}*c_{O_{2}}*\frac{c_{O_{2}}}{(c_{O_{2}}+c_{{NH}_{4}}*L)}$ | Rkn 8 : $k_{mox}*c_{Mn(II)}*C_{O_{2}}*\frac{c_{O_{2}}}{(c_{O_{2}}+c_{Mn(II)}*L)}$ |
| Rkn 9 : $k_{fox}*c_{Fe(II)}*c_{O_{2}}*\frac{c_{O_{2}}}{(c_{O_{2}}+c_{Fe(II)}*L)}$ | Rkn 10 : $k_{sox}*c_{S(-II)}*c_{O_{2}}*\frac{c_{O_{2}}}{(c_{O_{2}}+c_{S(-II)}*L)}$ |
| Rkn 11 : $k_{chox}*c_{{CH}_{4}}*c_{O_{2}}*\frac{c_{O_{2}}}{(c_{O_{2}}+c_{{CH}_{4}}*L)}*\frac{c_{{CH}_{4}}}{({c_{{CH}_{4}}+c}_{O_{2}}*L)}$ | Rkn 12 : $k_{nhmo}*c_{{NH}_{4}}*c_{mo\_1}*\frac{c_{mo\_1}}{(c_{mo-1}+c_{{NH}_{4}}*L)}$ |
| Rkn 13 : $k_{nhmx}*c_{{NH}_{4}}*c_{mo\_2}*\frac{c_{mo\_2}}{(c_{mo\_2}+c_{{NH}_{4}}*L)}$ | Rkn 14 : $k_{fmo}*c_{Fe(II)}*c_{mo\_1}*\frac{c_{mo\_2}}{(c_{mo\_2}+c_{Fe(II)}*L)}$ |
| Rkn 15 : $k_{fmx}*c_{Fe(II)}*c_{mo\_2}*\frac{c_{mo\_2}}{(c_{mo\_2}+c_{Fe(II)}*L)}$ | Rkn 16 : $k_{smo}*c_{S(-II)}*c_{mo\_1}*\frac{c_{mo\_1}}{(c_{mo\_1}+c_{S(-II)}*L)}$ |
| Rkn 17 : $k_{smx}*c_{S(-II)}*c_{mo\_2}*\frac{c_{mo\_2}}{(c_{mo\_2}+c_{S(-II)}*L)}$ | Rkn 18 : $k_{sfo}*c_{S(-II)}*c_{foh\_1}*\frac{c_{foh\_1}}{(c_{foh\_1}+c_{S(-II)}*L)}$ |
| Rkn 19 : $k_{sfx}*c_{S(-II)}*c_{foh\_2}*\frac{c_{foh\_2}}{(c_{foh\_2}+c_{S(-II)}*L)}$ | Rkn 20 : $k_{chso}*c_{{CH}_{4}}*c_{{SO}_{4}}*\frac{c_{{CH}_{4}}}{(c_{{CH}_{4}}+c_{{SO}_{4}}*L)}$ |
| Rkn 21 : $k_{mon}*c_{Mn(II)}*c_{{NO}_{3}}*\frac{c_{{NO}_{3}}}{(c_{{NO}_{3}}+c_{Mn(II)}*L)}$ | Rkn 22 : $k_{nhfo}*c_{{NH}_{4}}*c_{foh\_1}*\frac{c_{foh\_1}}{(c_{foh\_1}+c_{{NH}_{4}}*L)}$ |
| Rkn 23 : $k_{nhfx}*c_{{NH}_{4}}*c_{foh\_2}*\frac{c_{foh\_2}}{\left( c_{foh\_2}+c_{{NH}_{4}}*L \right)}$ |  |
| Mineral precipitation reactions : | |
| Rkn 24 : $k_{vivpre}* {(\Omega}_{viv}^{\alpha}-1) if \Omega_{viv} \geq1$ | |
| Rkn 25 : $k_{vivdis}*c_{vivianite}* {(1-\Omega}_{viv}^{\alpha}) if \Omega_{viv} <1$ with $\Omega_{viv}=\frac{{{[c}_{Fe(II)}]}^{3}*{{[c}_{{HPO}_{4}}]}^{2}}{k_{eqviv}} and \alpha\approx0.2$ | |
| Rkn 26 : $k_{sviv}*c_{S(-II)}*c_{vivianite}$ | |
| Rkn 27 : $k_{ironsulfidepre}*( \Omega_{fes}-1) if \Omega_{fes} \geq1$ | |
| Rkn 28 : $k_{ironsulfidedis}*c_{fes}*\left( 1-\Omega_{fes} \right)*\frac{c_{fes}}{(c_{fes}+L)} if \Omega_{fes} <1$ with $\Omega_{fes}=\frac{c_{Fe(II)}*c_{S(-II)}}{k_{eqironsulfide}*H^{+}}$ | |
| Rkn 29 : $k_{pyrpre}*c_{fes}*c_{S(-II)}*\frac{c_{fes}}{(c_{fes}+L)}$ | |
| Rkn 30 : $k_{mncarbonatepre}*( \Omega_{mnco3}-1) if \Omega_{mnco3} \geq1$ with $\Omega_{mnco3}=\frac{c_{Mn(II)}*c_{{HCO}_{3}^{-}}*k_{eqHCO3CO3}}{k_{eqmncarbonate}*H^{+}}$ | |
